# Upregulation of AK7 in ACC-VTA glutamatergic projections contributes to chronic inflammation pain in male mice

**DOI:** 10.1038/s44319-026-00940-z

**Published:** 2026-09-21

**Authors:** Jie Huang, Meng-Ge Li, Ru-Yu Yan, Zhen-Hua Xu, Chen-Hao Zhang, Ying Cao, Xiao-Dong Xu, Yong-Chang Li

**Affiliations:** 1 Department of Pharmacy, The Zhangjiagang Affiliated Hospital of Soochow University, Zhangjiagang, P. R. China; 2 Center of Translational Medicine, The Zhangjiagang Affiliated Hospital of Soochow University, Zhangjiagang, P. R. China; 3 Jiangsu Key Laboratory of Neuropsychiatric Diseases and Institute of Neuroscience, Soochow University, Suzhou, P. R. China

**Keywords:** Neuroscience

## Abstract

Chronic inflammation pain is a complex pathological condition involving multiple mechanisms in both the peripheral and central nervous systems. Growing evidence indicates that an imbalance in central nervous system function plays a crucial role in the progression of chronic pain, yet the underlying neural circuits and molecular targets remain largely unclear. Here we identify the anterior cingulate cortex (ACC) as a key mediator in chronic inflammation pain. Noxious stimulation significantly increases c-Fos expression and calcium activity in both the ACC and the ventral tegmental area (VTA). Upregulation of adenosine kinase 7 (AK7) in ACC glutamatergic neurons emerges as an important factor in pain pathogenesis: interfering with AK7 expression reduces neuronal activity in the ACC and VTA and alleviates pain-related behaviors, while overexpressing AK7 enhances neuronal activity in these regions and exacerbates pain. Further experiments demonstrate that modulating the activity of VTA glutamatergic neurons can correspondingly reverse the effects of AK7 manipulation on pain phenotypes. In summary, our findings demonstrate that upregulation of AK7 in ACC promotes chronic pain through the ACC-VTA neural circuit.

## Synopsis

Chronic inflammatory pain induces AK7 upregulation in the ACC, which enhances intrinsic excitability and excitatory synaptic transmission of ACC glutamatergic neurons causing aberrant activation of the ACC-VTA glutamatergic circuit.

- CFA-induced chronic inflammatory pain increases AK7 expression in the ACC.
- AK7 upregulation enhances both the intrinsic excitability and excitatory synaptic transmission of ACC glutamatergic neurons.
- Increased excitatory output from the ACC activates downstream VTA glutamatergic neurons through the ACC-VTA glutamatergic projection, promoting chronic inflammatory pain.

## Abstract (web summary)

Chronic inflammatory pain induces AK7 upregulation in the ACC, which enhances intrinsic excitability and excitatory synaptic transmission of ACC glutamatergic neurons causing aberrant activation of the ACC-VTA glutamatergic circuit.

## Graphical abstract

**Figure:**
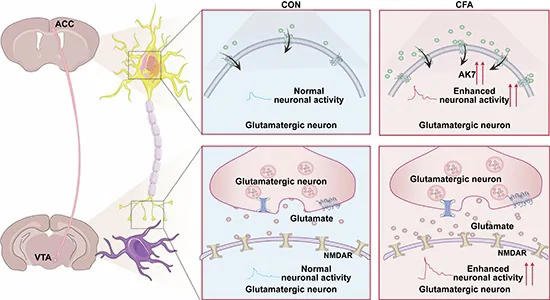

## Introduction

Chronic pain is defined as pain persisting for more than three months and is frequently accompanied by emotional disturbances and a reduced quality of life, thereby substantially impairing patients’ daily functioning and mental health (Cohen et al, 2021). It represents a complex pathological condition with pervasive effects across physiological, psychological, and social domains (Arango-Dávila and Rincón-Hoyos, 2018). Despite extensive research efforts, the management of chronic pain remains challenging, as conventional pharmacological therapies often provide limited efficacy and are associated with undesirable adverse effects (Treede et al, 2019). Complete Freund’s adjuvant (CFA) is a widely used experimental agent for inducing inflammation and pain. By activating immune responses and inflammatory signaling pathways, CFA produces sustained local inflammation and persistent pain at the injection site. CFA-based murine models effectively recapitulate key features of human chronic inflammatory disorders, such as rheumatoid arthritis, and have therefore become a standard tool in pain research (Liu et al, 2025; Wang and Li, 2024). In recent years, investigations using CFA-induced inflammatory pain models have primarily focused on elucidating the underlying molecular mechanisms and identifying novel therapeutic targets.

The pathogenesis of chronic inflammatory pain is highly complex and involves coordinated mechanisms within both the peripheral and central nervous systems (Salaffi et al, 2018). Peripherally, inflammatory mediators activate nociceptors, leading to neuronal sensitization and heightened pain perception—a phenomenon known as peripheral sensitization (Garrity et al, 2023; Zhang et al, 2021). Centrally, maladaptive processing and modulation of nociceptive signals contribute to the affective and cognitive dimensions of pain, including emotional distress and pain-related memory formation (Qi et al, 2024; Tassou et al, 2025). The brain plays a critical role in these processes, with the anterior cingulate cortex (ACC) emerging as a key structure involved in inflammatory pain (Franciosa et al, 2024; Gungor and Johansen, 2019; Habermann et al, 2025; Wei et al, 2024). The ACC, located in the medial frontal lobe, serves as a central hub for integrating emotional, cognitive, and nociceptive information (Li et al, 2024; Zhou et al, 2025). It exhibits a complex laminar architecture and extensive neural connectivity. The ACC is primarily composed of glutamatergic (Glu), and γ-aminobutyric acid (GABAergic), playing a dominant role in pain modulation and emotional regulation (Gungor and Johansen, 2019; Xue et al, 2024). Advances in neuroimaging techniques, particularly functional magnetic resonance imaging (fMRI), have enabled real-time assessment of ACC activity during inflammatory pain states (Abe et al, 2022). Notably, fMRI studies have consistently demonstrated pronounced ACC activation in patients experiencing inflammatory pain, with activation levels positively correlating with subjective pain intensity (Apkarian et al, 2005; Jiang et al, 2025).

Among the downstream targets of the ACC, the ventral tegmental area (VTA) has attracted increasing attention. Accumulating evidence indicates that the VTA participates in the regulation of pain perception, defensive behaviors, sleep–wake cycles, and motivational states (Barbano et al, 2020; Yu et al, 2019; Zhou et al, 2019). However, the role of the ACC-VTA neural circuit in mediating chronic inflammatory pain remains incompletely elucidated. Adenylate kinase 7 (AK7) is a phosphotransferase enzyme that plays an essential role in cellular energy homeostasis and signal transduction by catalyzing the reversible interconversion of adenine nucleotides—ATP, ADP, and AMP (Deline et al, 2021; Wu et al, 2024). This enzymatic activity is crucial for maintaining intracellular energy balance, particularly under conditions of metabolic stress or cellular challenge (Zhong et al, 2024). In the nervous system, AK7 is implicated not only in energy metabolism but also in the regulation of neuronal excitability and synaptic plasticity (Chen et al, 2015; Guclu et al, 2022). Emerging evidence suggests that adenylate kinases may indirectly modulate adenosine receptor activation by regulating adenosine availability or influence the balance of key neurotransmitters, such as dopamine and glutamate, thereby shaping synaptic transmission and neural circuit function (Dzeja and Terzic, 2009; Kaur and Goel, 2025). However, whether AK7 expressed in ACC glutamatergic neurons contributes to the central processing and modulation of chronic inflammatory pain remains unknown.

In the present study, we employed a CFA-induced mouse model of inflammatory pain and demonstrate that glutamatergic projections from the ACC to the VTA regulate CFA-evoked pain-related behaviors. Furthermore, we provide evidence that AK7 expression in ACC glutamatergic neurons plays a pivotal role in modulating this pain-related neural pathway. Collectively, these findings deepen our understanding of the central mechanisms underlying CFA-induced inflammatory pain and offer novel insights into potential therapeutic targets for chronic pain management.

## Results

### CFA-induced hyperalgesia in mice and activation of the ACC

**Figure 1:**
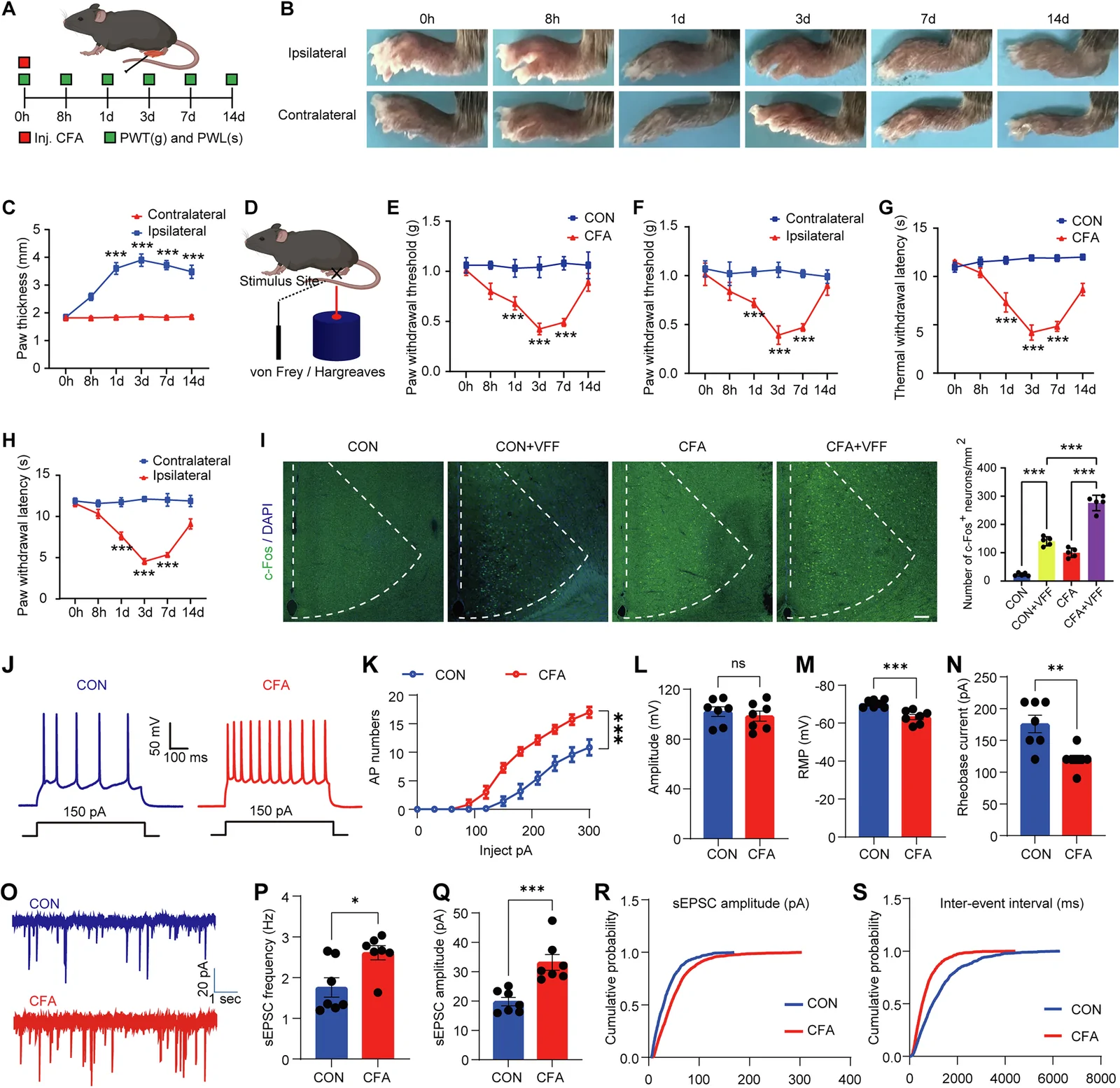
CFA induces persistent inflammatory hyperalgesia and activates the ACC. (**A**) Schematic illustration of the CFA-induced inflammatory pain model in mice. (**B**, **C**) Representative images and quantification of paw swelling following CFA injection, showing persistent inflammation lasting up to two weeks (***P < 0.0001, two-way ANOVA followed by Sidak’s multiple comparisons test, *n* = 5 mice per group). (**D**–**H**) PWT and PWL were significantly reduced 1 day after CFA injection compared with CON mice and contralateral paws, indicating successful establishment of inflammatory hyperalgesia (***P < 0.0001, two-way ANOVA followed by Sidak’s multiple comparisons test, *n* = 5 mice per group) (**I**) Representative images and quantification of c-Fos expression in the ACC following repeated von Frey stimulation. CFA-treated mice exhibited significantly increased c-Fos–positive neurons compared with CON mice (***P < 0.0001, one-way ANOVA with Tukey’s multiple-comparisons test, *n* = 5 brain sections from 5 mice per group). Scale = 100 µm. (**J**) Representative action potential (AP) firing traces elicited by 150 pA depolarizing current injection in ACC neurons of CON and CFA mice. Scale bars: 50 mV, 100 ms. (**K**) Input–output curve showing the number of evoked APs in response to gradient injected currents (0–300 pA) (***P < 0.0001, two-sample Student’s *t* test, *n *= 7 neurons per group). (**L**) Statistical analysis of AP peak amplitude between CON and CFA groups (*P* = 0.5404, no significant difference, two-sample Student’s *t* test, *n* = 7 neurons per group). (**M**) Quantitative summary of resting membrane potential (RMP) of ACC neurons in two groups (***P = 0.0003, two-sample Student’s *t* test, *n* = 7 neurons per group). (**N**) Statistical comparison of rheobase current required to trigger the first AP in CON and CFA groups (**P = 0.0033, two-sample Student’s *t* test, *n* = 7 neurons per group). (**O**) Representative raw traces of spontaneous excitatory postsynaptic currents (sEPSCs) recorded from ACC glutamatergic neurons in the CON (blue) and CFA (red) groups. Scale bars: 20 pA, 1 s. (**P**) Statistical comparison of sEPSCs frequency between the two groups (*P = 0.0138, two-sample Student’s *t* test, *n* = 7 neurons per group). (**Q**) Quantitative analysis of sEPSC amplitude in CON and CFA groups (***P = 0.0009, two-sample Student’s *t* test, *n* = 7 neurons per group). (**R**, **S**) Cumulative probability distribution curves of sEPSC amplitude (**R**) and inter-event interval (**S**) from two experimental groups. Scale bar = 100 µm. The data are represented as mean ± SEM.

A schematic illustration of the CFA-induced inflammatory pain model is shown in Fig. 1A. Injection of CFA into the left hind paw, with saline injected into the contralateral paw as a control, produced robust and persistent CFA-specific inflammation. Measurement of paw thickness revealed significant plantar swelling of the left hind paw persisted for up to two weeks following CFA administration (Fig. 1B,C, ***P < 0.001, two-way ANOVA followed by Sidak’s multiple comparisons test). One day after CFA injection, both paw withdrawal threshold (PWT) and paw withdrawal latency (PWL) were significantly reduced in the CFA-treated paw compared with the contralateral paw and with control (CON) mice (Fig. 1D–H, ***P < 0.001, two-way ANOVA followed by Sidak’s multiple comparisons test). These results confirm the successful establishment of an inflammatory pain model in adult mice. To determine whether the ACC is activated during inflammatory pain, mice were subjected to von Frey stimulation. Repeated mechanical stimulation markedly increased c-Fos expression in the ACC. Moreover, c-Fos expression in the ACC was significantly higher in CFA-treated mice than in CON mice (Fig. 1I, ***P < 0.001, one-way ANOVA with Tukey’s multiple-comparisons test), indicating robust ACC activation during CFA-induced inflammatory pain.

Furthermore, to directly assess functional alterations in intrinsic excitability and excitatory synaptic transmission of ACC neurons during chronic inflammatory pain, we performed ex vivo whole-cell patch-clamp recordings in acute ACC brain slices obtained from CON and CFA mice. Representative action potential (AP) firing traces evoked by a 150-pA current injection are shown in Fig. 1J. Compared with CON mice, ACC neurons from CFA mice exhibited significantly increased AP firing in response to identical current stimulation, as demonstrated by the enhanced input–output relationship (Fig. 1K, ***P < 0.001, two-sample Student’s *t* test). However, no significant difference was observed in AP peak amplitude between the two groups (Fig. 1L, ns, two-sample Student’s *t* test). Analysis of intrinsic membrane properties revealed that ACC neurons from CFA mice displayed a significantly depolarized resting membrane potential (RMP) (Fig. 1M, ***P < 0.001, two-sample Student’s *t* test) and a reduced rheobase current (Fig. 1N, **P < 0.01, two-sample Student’s *t* test), indicating enhanced intrinsic neuronal excitability. We next examined excitatory synaptic transmission by recording spontaneous excitatory postsynaptic currents (sEPSCs) from ACC neurons. Representative sEPSCs traces are shown in Fig. 1O. Quantitative analysis demonstrated that both the frequency (Fig. 1P, *P < 0.05, two-sample Student’s *t* test) and amplitude (Fig. 1Q, ***P < 0.001, two-sample Student’s *t* test) of sEPSCs were significantly increased in CFA mice compared with CON mice. Consistently, cumulative probability analysis revealed a leftward shift in inter-event interval distribution and an upward shift in amplitude distribution of sEPSCs in CFA neurons (Fig. 1R,S), suggesting enhanced excitatory synaptic transmission in the ACC following chronic inflammatory pain.

Together, these findings provide direct electrophysiological evidence that chronic inflammatory pain induces functional hyperexcitability of ACC neurons, characterized by increased intrinsic excitability and potentiated glutamatergic synaptic transmission. These findings establish ACC neuronal hyperexcitability as a key electrophysiological hallmark of chronic inflammatory pain and provide a functional basis for further investigating the molecular mechanisms underlying ACC dysfunction.

### CFA-induced activation of ACC glutamatergic neurons

**Figure 2:**
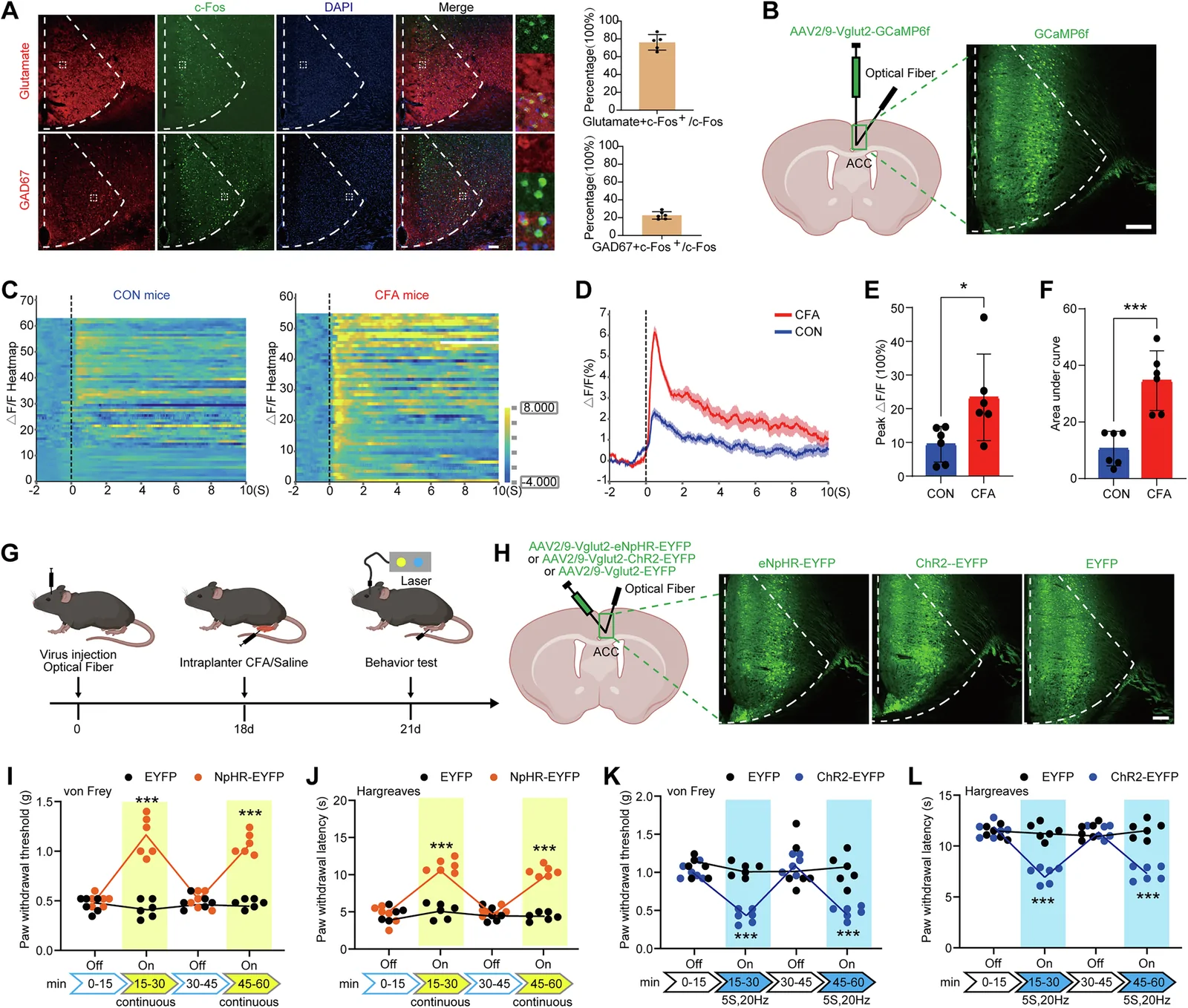
Activation of ACC glutamatergic neurons contributes to inflammatory pain. (**A**) Immunofluorescence staining showing colocalization of c-Fos with glutamatergic and GABAergic neurons in the ACC after CFA treatment. (**B**) Schematic of viral injection and fiber photometry recording of glutamatergic neurons in the ACC using AAV2/9-vglut2-GCaMP6f. (**C**–**F**) Representative calcium traces and quantitative analysis demonstrating increased Ca²⁺ activity of ACC glutamatergic neurons in CFA mice compared with CON mice following von Frey stimulation (*P = 0.0343, ***P = 0.0007, two-sample Student’s *t* test, *n* = 6 mice per group). (**G**) Experimental design for optogenetic manipulation of ACC glutamatergic neurons. (**H**) Representative images showing expression of eNpHR or ChR2 in ACC glutamatergic neurons. (**I**, **J**) Optogenetic inhibition of ACC glutamatergic neurons significantly increased PWT and PWL in CFA mice (***P < 0.0001, two-way ANOVA followed by Sidak’s multiple-comparisons test, *n* = 6 mice per group). (**K**, **L**) Optogenetic activation of ACC glutamatergic neurons decreased PWT and PWL in CON mice (***P < 0.0001, two-way ANOVA followed by Sidak’s multiple-comparisons test, *n* = 6 mice per group). Scale bar = 100 µm. The data are represented as mean ± SEM.

**Figure EV1:**
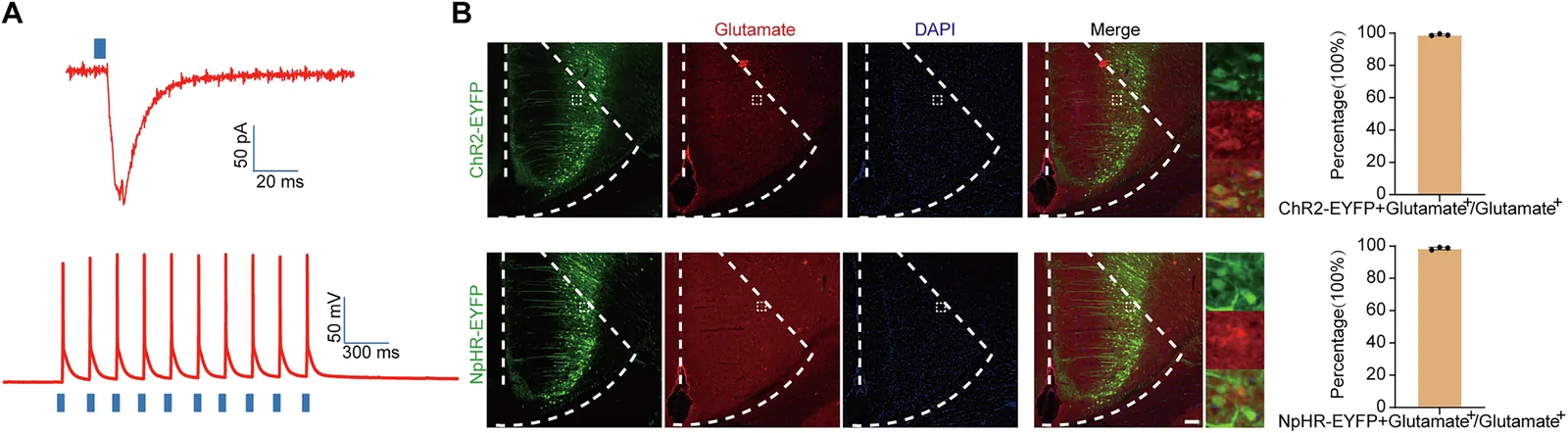
Functional validation and cell-type specificity of optogenetic viral expression in ACC glutamatergic neurons. (**A**) Ex vivo whole-cell patch-clamp recordings demonstrating the functional efficacy of ChR2 expressed in ACC glutamatergic neurons. Upper panel: Representative light-evoked inward photocurrent recorded from a ChR2-expressing neuron following brief blue light stimulation. Lower panel: Representative action potential firing induced by repetitive blue light pulses in a ChR2-expressing neuron. Scale bars: 50 pA, 20 ms (upper); 50 mV, 300 ms (lower). (**B**) Representative immunofluorescence images showing colocalization of ChR2-EYFP (upper panels) or NpHR-EYFP (lower panels) with the glutamatergic neuronal marker Glutamate in the ACC (*n* = 3 mice per group). Scale bar = 100 µm. The data are represented as mean ± SEM.

To identify the neuronal populations activated in the ACC during inflammatory pain, immunofluorescence staining was performed to co-label c-Fos with cell-type-specific markers. c-Fos-positive cells were predominantly colocalized with markers of glutamatergic neurons and, to a lesser extent, GABAergic neurons (Fig. 2A). These findings indicate that the majority of activated neurons in the ACC of CFA-treated mice are glutamatergic, suggesting a prominent role for excitatory neurons in CFA-induced ACC activation. To further validate the activation of glutamatergic neurons in the ACC, in vivo calcium dynamics were recorded in awake, freely behaving mice using fiber photometry. AAV2/9-Vglut2-GCaMP6f was stereotactically injected into the ACC to selectively label glutamatergic neurons (Fig. 2B). Following 1.0 g von Frey stimulation, Ca²⁺ activity in ACC glutamatergic neurons was significantly elevated in CFA-treated mice compared with CON mice (Fig. 2C–F, *P < 0.05, ***P < 0.001, two-sample Student’s *t* test). In addition, optogenetic approaches were employed to directly assess the causal role of ACC glutamatergic neurons in pain modulation (Fig. 2G). AAV2/9-Vglut2-eNpHR-EYFP, AAV2/9-Vglut2-ChR2-EYFP or AAV2/9-Vglut2- EYFP was injected into the ACC of CFA-treated or CON mice, respectively, to selectively inhibit or activate glutamatergic neurons (Fig. 2H). The electrophysiological and immunofluorescence results of the isolated brain slices confirmed the functional effectiveness and cell-targeting specificity of the light-sensitive protein (Fig. EV1).

Optogenetic inhibition of ACC glutamatergic neurons using yellow light significantly attenuated mechanical and thermal hypersensitivity in CFA-treated mice, as reflected by increased PWT and PWL (Fig. 2I,J, ***P < 0.001, two-way ANOVA followed by Sidak’s multiple-comparisons test). Conversely, optogenetic activation of ACC glutamatergic neurons using blue light significantly enhanced pain sensitivity in CON mice (Fig. 2K,L, ***P < 0.001, two-way ANOVA followed by Sidak’s multiple-comparisons test). Importantly, light stimulation in mice expressing control viruses did not alter pain-related behaviors, confirming the specificity of the optogenetic manipulations. Collectively, these results demonstrate that ACC glutamatergic neurons are critically involved in the regulation of CFA-induced inflammatory pain.

### The ACC-VTA glutamatergic projection regulates inflammatory pain behavior

**Figure 3:**
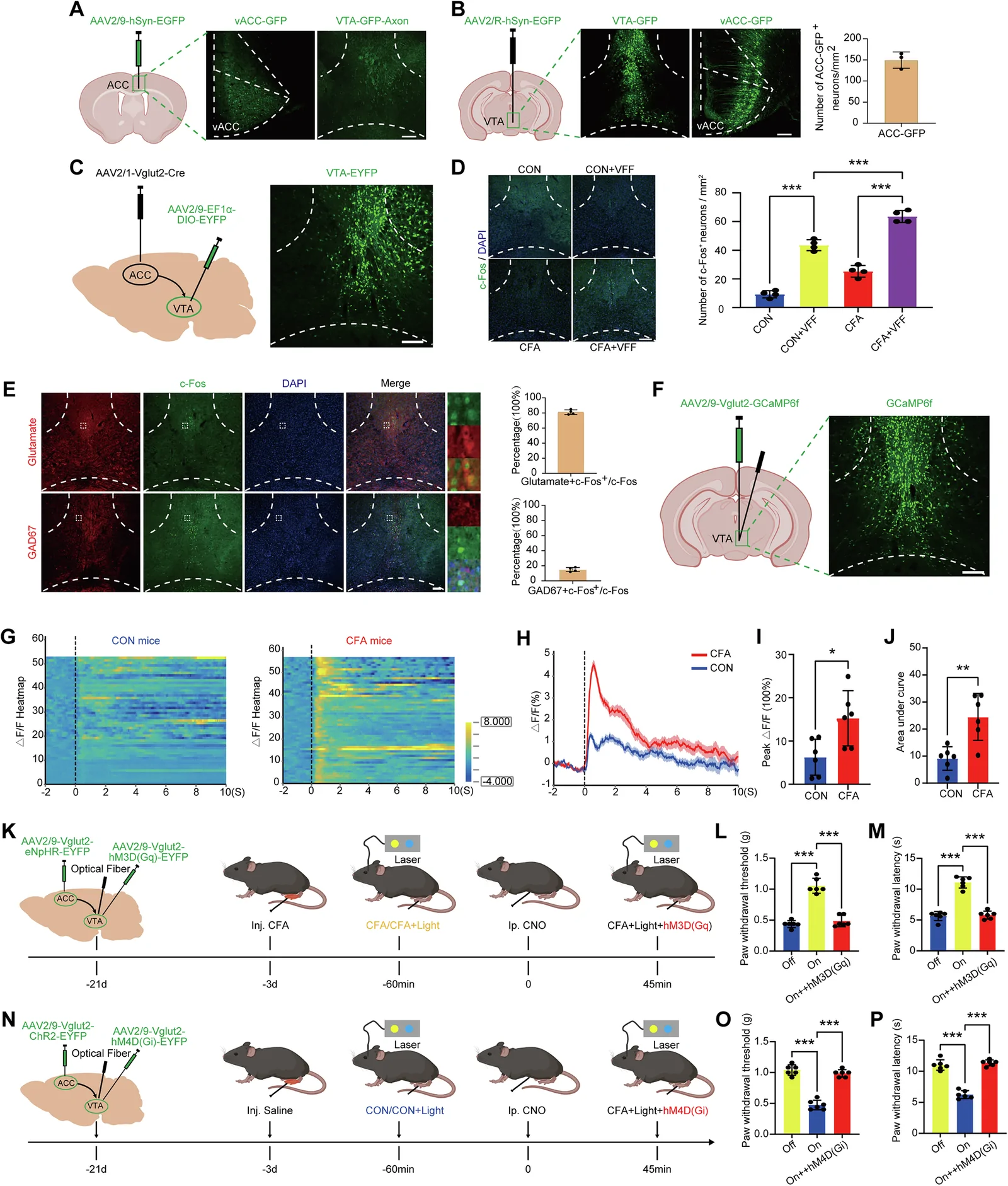
The ACC-VTA glutamatergic projection regulates inflammatory pain. (**A**) Anterograde viral tracing from the ACC showing dense axonal projections to the VTA. (**B**) Retrograde viral tracing from the VTA confirming projection neurons located in the ACC. (**C**) Dual-virus strategy demonstrating glutamatergic projections from ACC to VTA. (**D**) Representative images and quantification of c-Fos expression in the VTA after repeated von Frey stimulation (***P < 0.0001, one-way ANOVA with Tukey’s multiple-comparisons test, *n* = 4 brain sections from 4 mice per group). (**E**) Immunofluorescence showing colocalization of c-Fos with glutamatergic and GABAergic neurons in the VTA. (**F**) Schematic of fiber photometry recording of VTA glutamatergic neurons. (**G**–**J**) Calcium imaging showing enhanced Ca²⁺ activity of VTA glutamatergic neurons in CFA mice (*P = 0.0162, **P = 0.003, two-sample Student’s *t* test, *n* = 6 mice per group). (**K**–**M**) Dual-virus strategy combining optogenetic inhibition of ACC-VTA terminals and chemogenetic activation of VTA glutamatergic neurons (***P < 0.0001, one-way ANOVA with Tukey’s multiple-comparisons test, *n* = 6 mice per group). (**N**–**P**) Complementary manipulation in CON mice showing that optogenetic activation of ACC-VTA projections induced hyperalgesia, which was reversed by chemogenetic inhibition of VTA glutamatergic neurons (***P < 0.0001, one-way ANOVA with Tukey’s multiple-comparisons test, *n* = 6 mice per group). Scale bar = 100 µm. The data are represented as mean ± SEM.

**Figure EV2:**
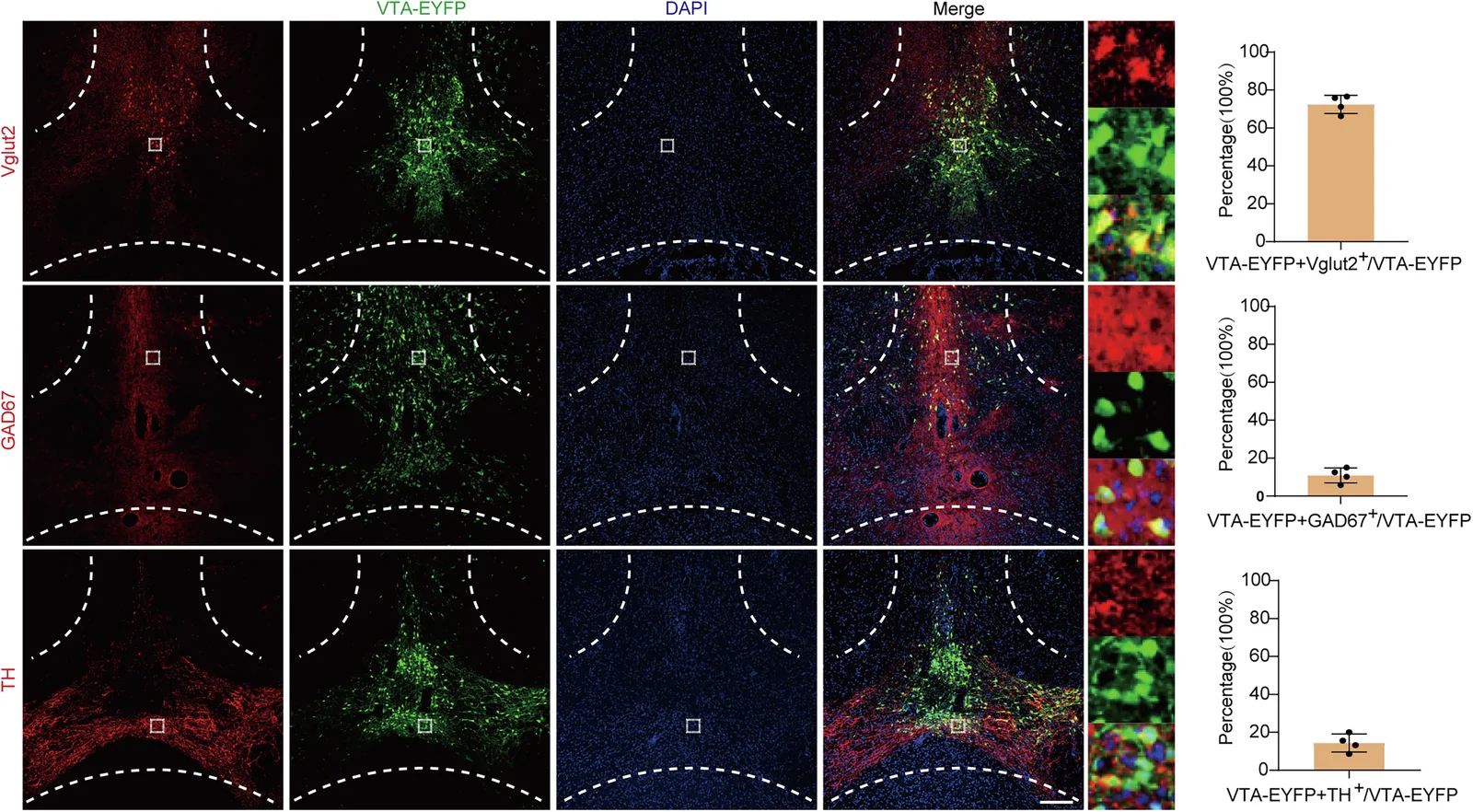
Identification of the major postsynaptic neuronal population receiving ACC glutamatergic projections in the VTA. A dual-virus anterograde tracing strategy was used to label ACC-derived EYFP-positive projections in the VTA, followed by immunofluorescence colocalization analysis with markers for the three major neuronal populations in the VTA: VGLUT2 (glutamatergic neurons, upper panels), GAD67 (GABAergic neurons, middle panels), and TH (dopaminergic neurons, lower panels) (*n* = 4 mice per group). Scale bar = 100 µm. The data are represented as mean ± SEM.

Our preceding experiments demonstrated that glutamatergic neurons in the ACC play a critical role in regulating inflammatory pain behavior in mice. To identify downstream brain regions through which the ACC exerts this regulatory function, we employed viral tracing strategies to map ACC efferent projections. An anterograde tracer virus, AAV2/9-hSyn-EGFP, was stereotactically injected into the ACC. Twenty-one days after injection, abundant EGFP-labeled axon terminals were observed in the VTA (Fig. 3A), indicating a robust anterograde projection from the ACC to the VTA. To further confirm this connectivity, a retrograde tracer virus, AAV2/R-hSyn-EGFP, was injected into the VTA, resulting in strong retrogradely labeled neuronal soma in the ACC (Fig. 3B), and these retrograde-marked neuron cell bodies are mainly concentrated in the vACC subregion of the ACC. Together, these results confirm a direct anatomical projection from the ACC to the VTA. Given the prominent involvement of glutamatergic neurons in inflammatory pain, we next tested whether ACC glutamatergic neurons project to the VTA. To this end, AAV2/1-Vglut2-Cre was injected into the ACC to selectively express Cre recombinase in glutamatergic neurons, while AAV2/9-EF1α-DIO-EYFP was simultaneously injected into the VTA. Robust EYFP fluorescence was detected in the VTA (Fig. 3C). In addition, these EYFP fluorescence mainly co-localizes with glutamatergic neurons (Fig. EV2). These results provide direct evidence for an anterograde glutamatergic projection from the ACC to the VTA.

**Figure EV3:**
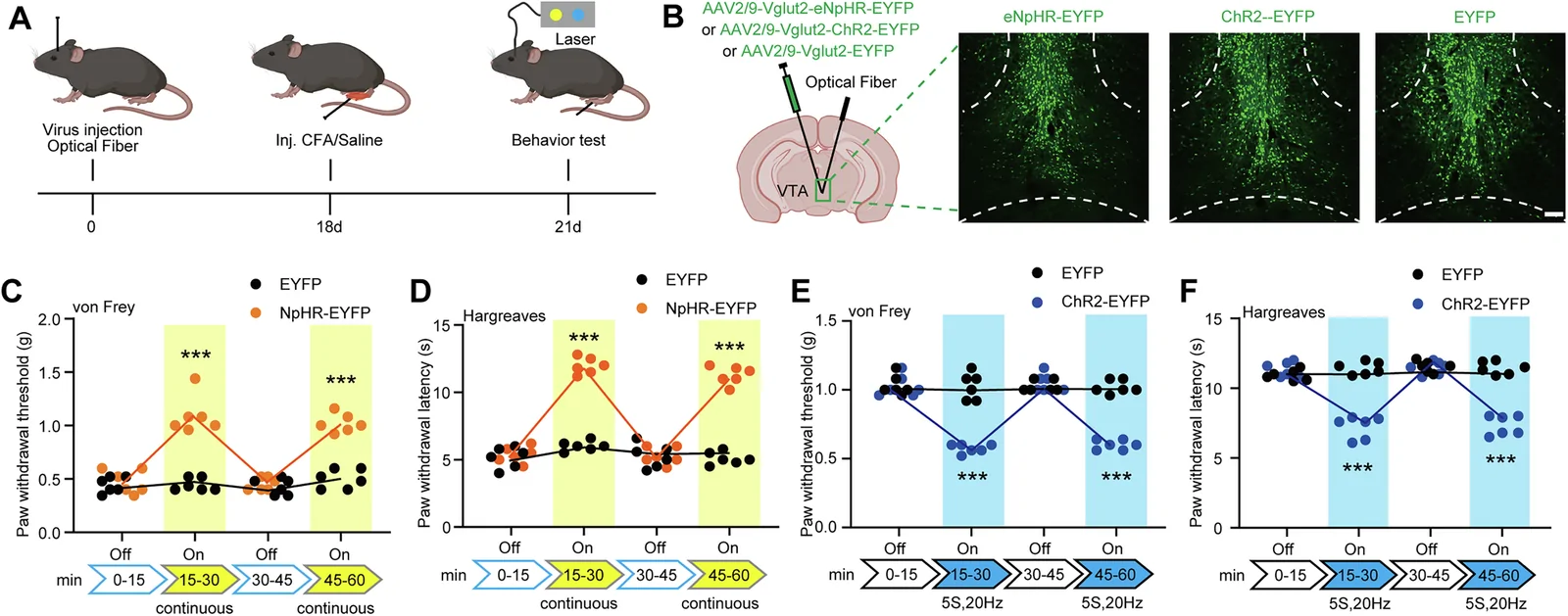
Optogenetic manipulation of VTA glutamatergic neurons alters pain behavior. (**A**) Experimental design for optogenetic manipulation of VTA glutamatergic neurons. (**B**) Representative images showing expression of eNpHR or ChR2 in VTA glutamatergic neurons. (**C**, **D**) Optogenetic inhibition of VTA glutamatergic neurons significantly increased PWT and PWL in CFA mice (***P < 0.0001, two-way ANOVA followed by Sidak’s multiple-comparisons test, *n* = 6 mice per group). (**E**, **F**) Optogenetic activation of VTA glutamatergic neurons decreased PWT and PWL in CON mice (***P < 0.0001, two-way ANOVA followed by Sidak’s multiple-comparisons test, *n* = 6 mice per group). Scale bar = 100 µm. The data are represented as mean ± SEM.

We next examined whether inflammatory pain also activates the VTA. Following repeated von Frey stimulation, c-Fos expression in the VTA was significantly increased, with CFA-treated mice exhibiting markedly higher c-Fos levels than CON mice (Fig. 3D, ***P < 0.001, one-way ANOVA with Tukey’s multiple-comparisons test). To identify the neuronal populations activated in the VTA, immunofluorescence staining was performed to co-label c-Fos with neuronal subtype markers. c-Fos-positive cells were extensively colocalized with glutamatergic and, to a lesser extent, GABAergic neurons (Fig. 3E), indicating that glutamatergic neurons represent the predominant activated population in the VTA during CFA-induced inflammatory pain. To further validate these findings, we performed in vivo fiber photometry to record calcium activity in VTA glutamatergic neurons. AAV2/9-Vglut2-GCaMP6f was stereotactically injected into the VTA to selectively label glutamatergic neurons (Fig. 3F). Following von Frey stimulation, Ca²⁺ activity in VTA glutamatergic neurons was significantly elevated in CFA-treated mice compared with CON mice (Fig. 3G–J, *P < 0.05, **P < 0.01, two-sample Student’s *t* test). Consistent with these observations, optogenetic manipulation of VTA glutamatergic neurons significantly altered CFA-induced pain behaviors (Fig. EV3, ***P < 0.001, two-way ANOVA followed by Sidak’s multiple-comparisons test), further supporting a functional role for these neurons in inflammatory pain modulation.

Although both ACC and VTA glutamatergic neurons were shown to participate in inflammatory pain processing and were anatomically connected, the functional contribution of the ACC-VTA glutamatergic pathway to CFA-induced pain remained unclear. To address this, we employed a dual-virus strategy to selectively manipulate this circuit. In CFA-treated mice, AAV2/9-Vglut2-eNpHR-EYFP was injected into the ACC, while AAV2/9-Vglut2-hM3Dq-EYFP was injected into the VTA to chemogenetically activate VTA glutamatergic neurons. An optical fiber was implanted above the VTA to allow selective inhibition of ACC-derived glutamatergic axon terminals (Fig. 3K). Baseline PWT and PWL were first recorded under light-off conditions. Optogenetic inhibition of ACC-VTA glutamatergic terminals significantly alleviated CFA-induced hyperalgesia. Notably, subsequent chemogenetic activation of VTA glutamatergic neurons with CNO reversed this analgesic effect (Fig. 3L,M, ***P < 0.001, one-way ANOVA with Tukey’s multiple-comparisons test), indicating that VTA glutamatergic neurons act downstream of ACC inputs to regulate inflammatory pain behavior. Conversely, in CON mice, AAV2/9-Vglut2-ChR2-EYFP was injected into the ACC, while AAV2/9-Vglut2-hM4Di-EYFP was injected into the VTA to chemogenetically inhibit VTA glutamatergic neurons (Fig. 3N). Optogenetic activation of ACC-VTA glutamatergic terminals significantly exacerbated pain behaviors in CON mice. Importantly, chemogenetic inhibition of VTA glutamatergic neurons with CNO abolished this pro-nociceptive effect (Fig. 3O,P, ***P < 0.001, one-way ANOVA with Tukey’s multiple-comparisons test).

Collectively, these findings demonstrate that the ACC-VTA glutamatergic neural circuit plays a pivotal role in the initiation and maintenance of CFA-induced hyperalgesia and constitutes a key central pathway mediating inflammatory pain in mice.

### AK7 expression is significantly upregulated in ACC glutamatergic neurons of CFA-treated mice

**Figure 4:**
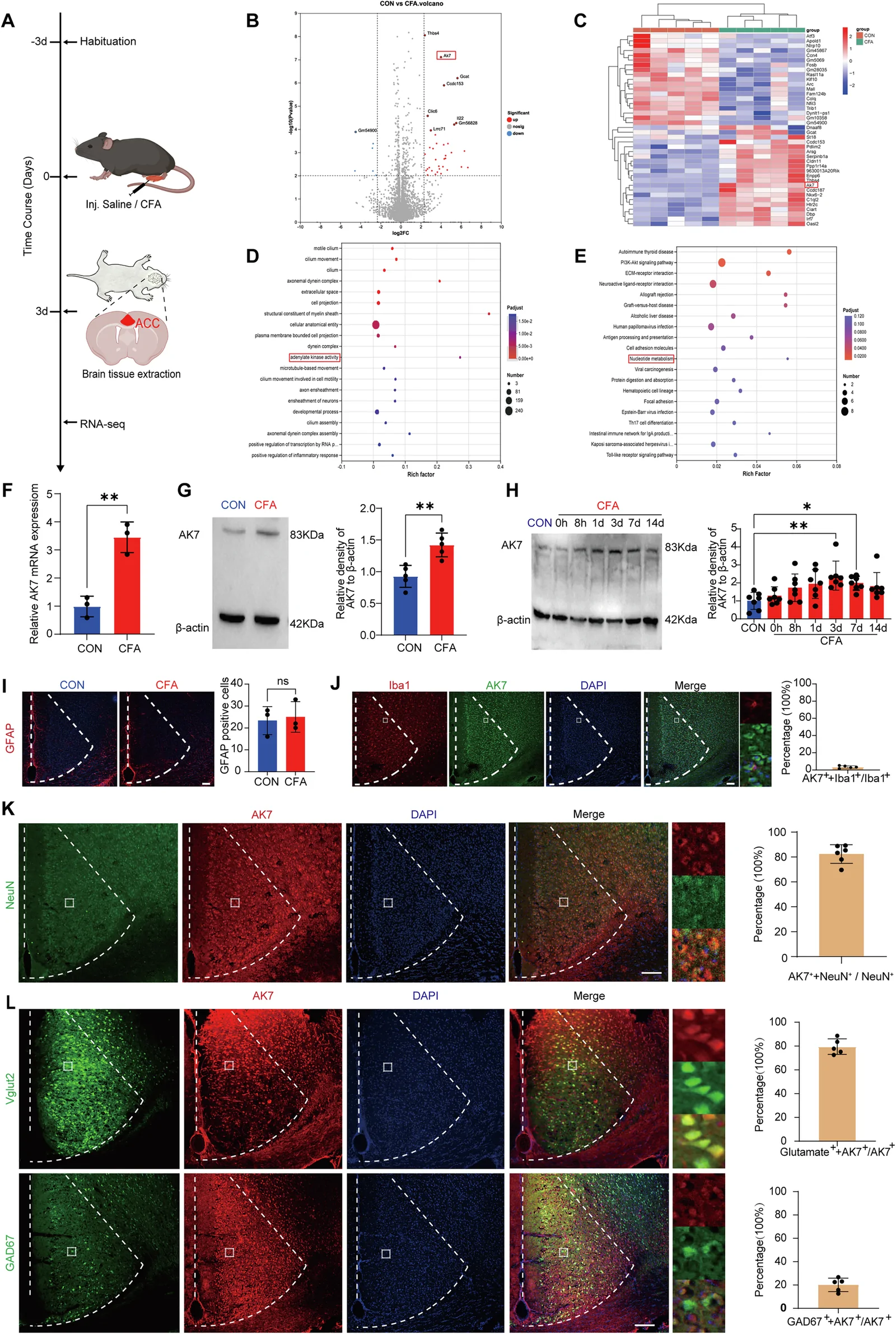
AK7 is selectively upregulated in ACC glutamatergic neurons of CFA mice. (**A**) Workflow for transcriptomic analysis of ACC tissue from CFA and CON mice. (**B**, **C**) Volcano plot and heatmap showing differentially expressed genes in the ACC following CFA treatment (Genes were considered differentially expressed if they met the following criteria: |log₂ fold change (FC)| ≥1 and *P* ≤ 0.05; Wald test, *n* = 5 mice per group). (**D**, **E**) Pathway enrichment analysis highlighting adenylate kinase and nucleotide metabolism–related signaling (Genes were considered differentially expressed if they met the following criteria: |log₂ fold change (FC)| ≥1 and *P* ≤ 0.05; Hypergeometric test). (**F**) RT-qPCR confirming upregulation of AK7 mRNA in the ACC of CFA mice (**P = 0.0029, two-sample Student’s *t* test, *n* = 3 mice per group). (**G**) Western blot confirming upregulation of AK7 protein in the ACC of CFA mice (**P = 0.0025, two-sample Student’s *t* test, *n* = 5 mice per group). (**H**) Western blot confirmed the expression of AK7 protein in the ACC region of CFA mice at different time points (*P = 0.0399, **P = 0.0017, one-way ANOVA with Tukey’s multiple-comparisons test, *n* = 7 mice per group). (**I**) Immunofluorescence showed the expression of astrocytes in the ACC of CON and CFA mice. (*P* = 0.7765, no significant difference, two-sample Student’s *t* test, *n *= 3 mice per group). (**J**) Immunofluorescence showed the localization of AK7 in microglia cells of ACC. (**K**) Immunofluorescence showing neuronal localization of AK7 in the ACC. (**L**) The colocalization of AK7 with glutamatergic neurons and GABAergic neurons in the ACC. Scale bar = 100 µm. The data are represented as mean ± SEM.

To further investigate the molecular mechanisms underlying CFA-induced inflammatory pain, we performed transcriptome sequencing to compare gene expression profiles in the ACC of CFA-treated and CON mice (Fig. 4A). Differential expression analysis revealed substantial transcriptional alterations in the ACC following CFA treatment. As illustrated by the volcano plot (Fig. 4B) and heatmap (Fig. 4C), 84 mRNA transcripts were significantly upregulated and 43 were significantly downregulated in CFA mice compared with CON mice. Pathway enrichment analysis indicated that adenylate kinase and nucleotide metabolism–related signaling pathways were among the most prominently altered in CFA-treated mice (Fig. 4D,E). Given the established role of adenylate kinases in neuronal energy metabolism and synaptic function (Zhong et al, 2024). We hypothesized that adenylate kinase 7 (AK7) may be a key regulator of CFA-induced pain signaling. Consistent with this hypothesis, Western blotting and RT-qPCR analyses confirmed that AK7 protein and mRNA levels were significantly upregulated in the ACC of CFA mice (Fig. 4F,G, **P < 0.01, two-sample Student’s *t* test). Notably, the temporal expression pattern of AK7 protein in the ACC of mice was closely associated with the progression of inflammatory pain behaviors (Fig. 4H, *P < 0.05, **P < 0.01, one-way ANOVA with Tukey’s multiple-comparisons test). To identify the cellular populations through which AK7 may contribute to pain sensitization, we next examined the expression pattern and cellular localization of AK7 in the ACC during chronic pain. Immunofluorescence analysis revealed that chronic pain induction did not result in apparent activation of astrocytes in the ACC (Fig. 4I). Moreover, AK7 showed minimal colocalization with microglial markers, suggesting that microglia are unlikely to be the primary cellular population expressing AK7 in the ACC during chronic pain (Fig. 4J). In contrast, AK7 was predominantly expressed in NeuN-positive neurons (Fig. 4K), indicating that neuronal AK7 may contribute to ACC dysfunction during chronic pain. Notably, AK7 showed extensive colocalization with glutamatergic neurons, indicating that excitatory neurons represent the primary cellular population exhibiting AK7 upregulation in CFA-treated mice (Fig. 4L). Together, these results suggest that selective upregulation of AK7 in ACC glutamatergic neurons may contribute to the development of inflammatory pain.

### AK7 expression in the ACC glutamatergic neurons regulates inflammatory pain behavior

**Figure 5:**
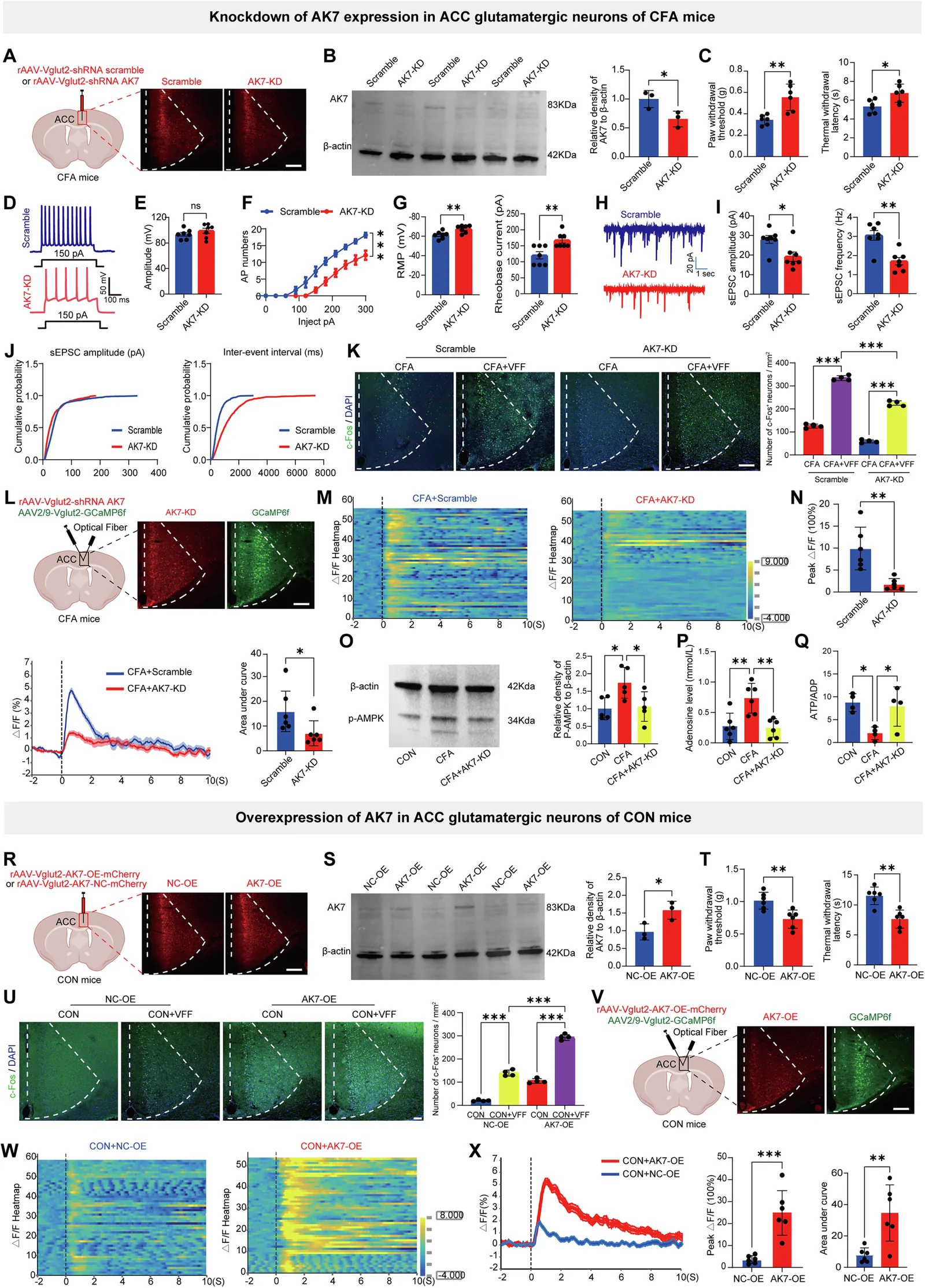
AK7 in ACC glutamatergic neurons regulates inflammatory pain. (**A**) Schematic of AK7 knockdown in ACC glutamatergic neurons using shRNA. (**B**) Western blot confirming effective AK7 knockdown in the ACC (*P = 0.0407, two-sample Student’s *t* test, *n* = 3 mice per group). (**C**) Knockdown of AK7 significantly increased PWT and PWL in CFA mice (*P = 0.0154, **P = 0.0028, two-sample Student’s *t* test, *n* = 6 mice per group). (**D**) Representative action potential firing traces elicited by 150 pA depolarizing current injection. Scale bars: 50 mV, 100 ms. (**E**) Statistical comparison of AP peak amplitude between the two groups (*P* = 0.2986, no significant difference, two-sample Student’s *t* test, *n* = 7 neurons per group). (**F**) Input–output curve showing the number of evoked APs under gradient injected currents (0–300 pA) (***P < 0.0001, two-way repeated-measures ANOVA). (**G**) Quantitative statistics of resting membrane potential (**P = 0.0087, two-sample Student’s *t* test, *n* = 7 neurons per group) and rheobase current (**P = 0.0067, two-sample Student’s *t* test, *n* = 7 neurons per group) of ACC neurons. (**H**) Representative raw traces of sEPSCs recorded from ACC neurons in Scramble (blue) and AK7-KD (red) groups. Scale bars: 20 pA, 1 s. (**I**) Statistical analysis of sEPSCs amplitude (left, *P = 0.0153, two-sample Student’s *t* test, *n* = 7 neurons per group) and frequency (right, **P = 0.0011, two-sample Student’s *t* test, *n* = 7 neurons per group) between two groups. (**J**) Cumulative probability distribution curves of sEPSCs amplitude (left) and inter-event interval (right) in Scramble and AK7-KD groups. (**K**) Immunofluorescence showing reduced c-Fos expression in the ACC after AK7 knockdown (***P < 0.0001, one-way ANOVA with Tukey’s multiple-comparisons test, *n* = 4 brain sections from 4 mice per group). (**L**–**N**) Fiber photometry showing decreased Ca²⁺ activity in ACC neurons following AK7 knockdown (*P = 0.0481, **P = 0.0027, two-sample Student’s *t* test, *n* = 6 per group). (**O**) The Western blot experiment confirmed the expression of p-AMPK protein in the ACC brain region of mice (*P = 0.0299; 0.0469, one-way ANOVA with Tukey’s multiple-comparisons test, *n* = 5 mice per group). (**P**) Biochemical quantitative detection results of adenosine concentration in the ACC brain region of mice (**P = 0.0042; 0.0027, one-way ANOVA with Tukey’s multiple-comparisons test, *n* = 6 mice per group). (**Q**) Biochemical quantitative detection results of ATP/ADP ratio in the ACC area of mice brain (*P = 0.0215; 0.0413, one-way ANOVA with Tukey’s multiple-comparisons test, *n* = 4 mice per group). (**R**) Schematic of AK7 overexpression in ACC glutamatergic neurons. (**S**) Western blot confirming AK7 overexpression (*P = 0.0364, two-sample Student’s *t* test, *n* = 4 mice per group). (**T**) Overexpression of AK7 decreased PWT and PWL in CON mice (**P = 0.0046; 0.0011, two-sample Student’s *t* test, *n* = 6 mice per group). (**U**) Increased ACC c-Fos expression following AK7 overexpression (***P < 0.0001, one-way ANOVA with Tukey’s multiple-comparisons test, *n* = 4 brain sections from 4 mice per group). (**V**–**X**) Fiber photometry showing enhanced Ca²⁺ activity in the ACC after AK7 overexpression (**P = 0.0052, ***P = 0.005, two-sample Student’s *t* test, *n* = 6 per group). Scale bar = 100 µm. The data are represented as mean ± SEM.

**Figure EV4:**
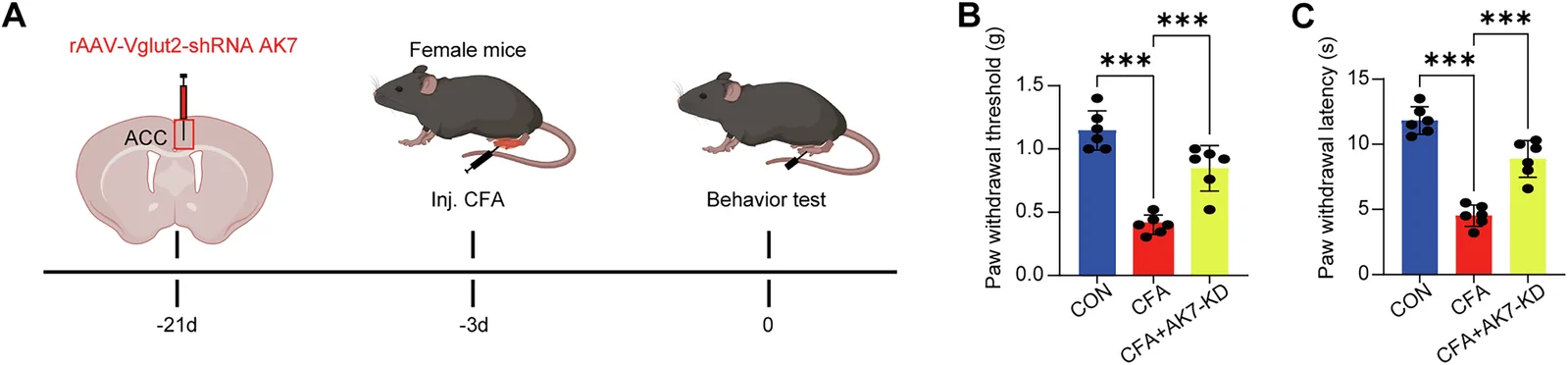
AK7 knockdown in ACC glutamatergic neurons attenuates CFA-induced mechanical and thermal hypersensitivity in female mice. (**A**) Schematic illustration of stereotaxic viral delivery for selective knockdown of AK7 in ACC glutamatergic neurons of female mice. (**B**, **C**) Knockdown of AK7 significantly increased PWT and PWL in CFA mice (***P < 0.0001, one-way ANOVA with Tukey’s multiple-comparisons test, *n* = 6 mice per group). The data are represented as mean ± SEM.

To determine the functional role of AK7 in inflammatory pain, we selectively knockdown AK7 expression in ACC glutamatergic neurons of CFA-treated mice by stereotactic injection of rAAV-Vglut2-shRNA AK7 (Fig. 5A). Western blot analysis confirmed a significant reduction of AK7 expression in the ACC following viral-mediated knockdown (Fig. 5B, *P < 0.05, two-sample Student’s *t* test). Behavioral analyses demonstrated that AK7 knockdown significantly alleviated CFA-induced hyperalgesia, as evidenced by increased PWT and PWL compared with scramble group (Fig. 5C, *P < 0.05, **P < 0.01, two-sample Student’s *t* test). A comparable analgesic effect was also observed in female mice, indicating that the protective effect of AK7 knockdown was not sex-specific (Fig. EV4).

To determine whether these behavioral improvements were accompanied by changes in neuronal function, ex vivo whole-cell patch-clamp recordings were performed in ACC neurons. Representative AP traces evoked by a 150-pA current injection are shown in Fig. 5D. Although AP peak amplitude remained unchanged between the two groups (Fig. 5E, ns, two-sample Student’s *t* test), AK7 knockdown significantly reduced the number of evoked APs across graded current injections (Fig. 5F, ***P < 0.001, two-sample Student’s *t* test). In addition, neurons from the AK7-knockdown group exhibited a more hyperpolarized RMP and a higher rheobase current than those in the scramble group (Fig. 5G, **P < 0.01, two-sample Student’s *t* test), indicating reduced intrinsic neuronal excitability. We next examined excitatory synaptic transmission by recording sEPSCs. Representative traces are shown in Fig. 5H. Quantitative analysis demonstrated that both the frequency and amplitude of sEPSCs were significantly decreased following AK7 knockdown (Fig. 5I, *P < 0.05, **P < 0.01, two-sample Student’s *t* test). Consistently, cumulative probability analysis of sEPSCs amplitude and inter-event intervals further supported the attenuation of enhanced excitatory synaptic transmission induced by chronic inflammatory pain (Fig. 5J).

Furthermore, repeated von Frey stimulation markedly increased c-Fos expression in the ACC of CFA mice; however, this effect was significantly attenuated following AK7 knockdown (Fig. 5K, ***P < 0.001, one-way ANOVA with Tukey’s multiple-comparisons test). In vivo fiber photometry further revealed that Ca²⁺ activity in ACC glutamatergic neurons was significantly reduced after AK7 knockdown in CFA mice (Fig. 5L–N, *P < 0.05, **P < 0.01, two-sample Student’s *t* test). These findings indicate that AK7 is required for maintaining heightened neuronal activity and pain hypersensitivity in the ACC during inflammatory pain.

To explore the underlying mechanism, we next examined key indicators of cellular energy metabolism in the ACC, including phosphorylated AMPK (p-AMPK), adenosine levels, and the ATP/ADP ratio. Compared with the CON group, CFA mice exhibited increased p-AMPK expression, elevated adenosine levels, and a reduced ATP/ADP ratio, indicating disrupted cellular energy homeostasis. Notably, all of these alterations were significantly ameliorated by AK7 knockdown (Fig. 5O–Q, *P < 0.05, **P < 0.01, one-way ANOVA with Tukey’s multiple-comparisons test). These findings suggest that AK7 promotes chronic inflammatory pain, at least in part, by disrupting energy metabolism in the ACC, thereby enhancing neuronal excitability and glutamatergic synaptic transmission.

To further validate the role of AK7, we selectively overexpressed AK7 in ACC glutamatergic neurons of CON mice by injecting rAAV-vglut2-AK7-P2A-mCherry (Fig. 5R). Western blot analysis confirmed successful AK7 overexpression in the ACC (Fig. 5S, *P < 0.05, two-sample Student’s *t* test). Behaviorally, AK7 overexpression significantly decreased PWT and PWL in CON mice, indicating the induction of pain hypersensitivity in the absence of peripheral inflammation (Fig. 5T, **P < 0.01, two-sample Student’s *t* test). Correspondingly, c-Fos expression in the ACC was significantly elevated following repeated von Frey stimulation in AK7-overexpressing mice compared with controls (Fig. 5U, ***P < 0.001, one-way ANOVA with Tukey’s multiple-comparisons test). Fiber photometry recordings further demonstrated increased Ca²⁺ activity in ACC glutamatergic neurons following AK7 overexpression (Fig. 5V–X, **P < 0.01, ***P < 0.001, two-sample Student’s *t* test). Collectively, these results indicate that AK7 expression in ACC glutamatergic neurons bidirectionally regulates inflammatory pain sensitivity.

### AK7 mediates inflammatory pain behavior via the ACC-VTA glutamatergic pathway

**Figure 6:**
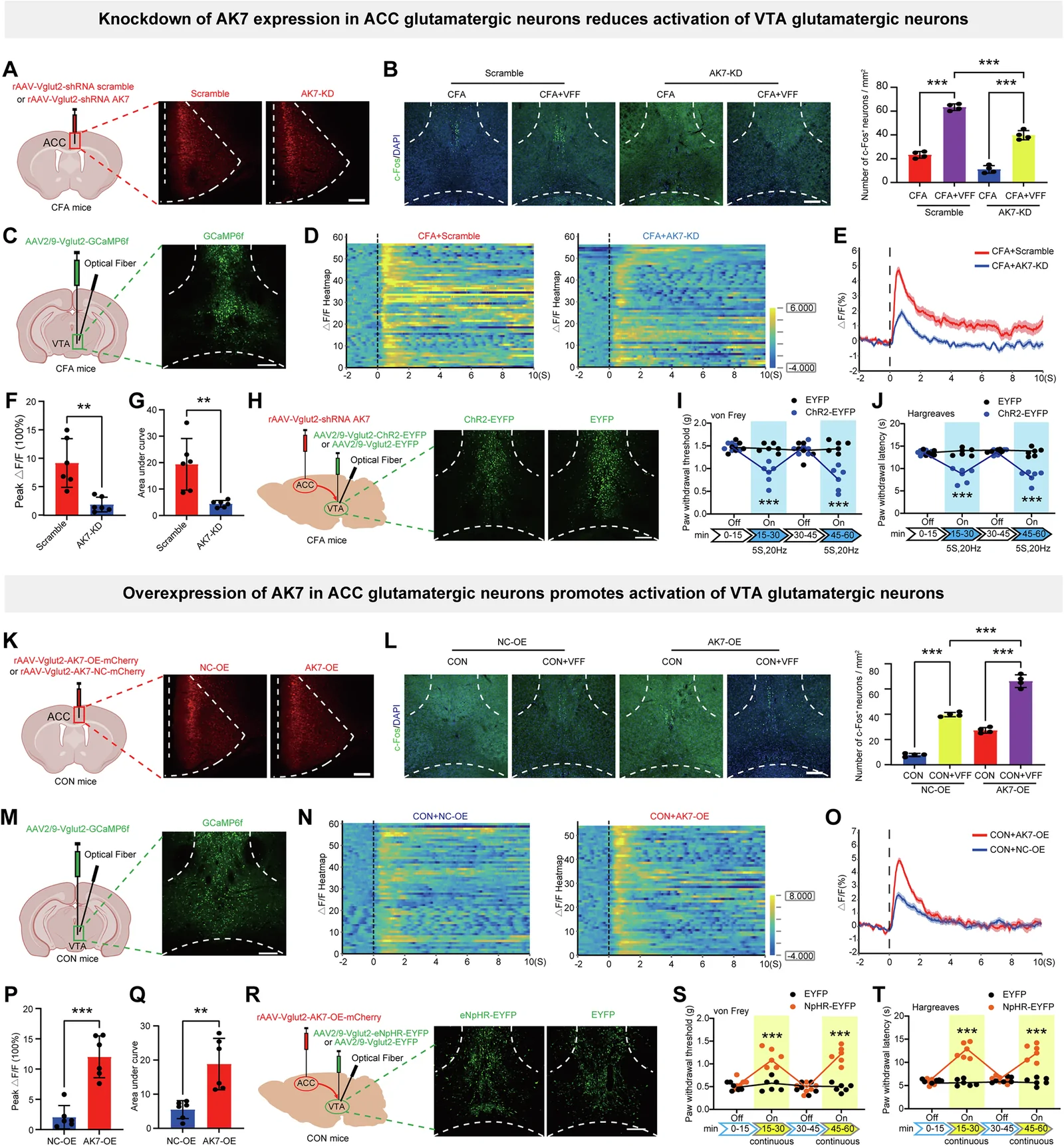
AK7 in the ACC modulates VTA activity and pain behavior. (**A**) Experimental design for assessing downstream VTA activity following AK7 manipulation in the ACC. (**B**) Reduced c-Fos expression in the VTA after AK7 knockdown in the ACC of CFA mice (***P < 0.0001, one-way ANOVA with Tukey’s multiple-comparisons test, *n* = 4 brain sections from 4 mice per group). (**C**–**G**) Fiber photometry showing decreased Ca²⁺ activity in VTA glutamatergic neurons after ACC AK7 knockdown (**P = 0.0025; 0.0040, two-sample Student’s *t* test, *n* = 6 mice per group). (**H**–**J**) Optogenetic activation of VTA glutamatergic neurons reversed the analgesic effect induced by ACC AK7 knockdown (***P < 0.0001, two-way ANOVA followed by Sidak’s multiple-comparisons test, *n* = 6 mice per group). (**K**) Schematic of ACC AK7 overexpression. (**L**) Increased VTA c-Fos expression following AK7 overexpression in the ACC (***P < 0.0001, one-way ANOVA with Tukey’s multiple-comparisons test, *n* = 4 brain sections from 4 mice per group). (**M**–**Q**) Elevated Ca²⁺ activity in VTA glutamatergic neurons after ACC AK7 overexpression (**P = 0.0021, ***P = 0.0001, two-sample Student’s *t* test, *n* = 6 mice per group). (**R**–**T**) Optogenetic inhibition of VTA glutamatergic neurons reversed AK7 overexpression-induced hyperalgesia (***P < 0.0001, two-way ANOVA followed by Sidak’s multiple-comparisons test, *n* = 6 mice per group). Scale bar = 100 µm. The data are represented as mean ± SEM.

Given the established ACC-VTA glutamatergic projection, we next examined whether AK7-mediated modulation of ACC activity influences downstream neuronal activity in the VTA. Following AK7 knockdown in the ACC of CFA-treated mice, the density of c-Fos–positive neurons in the VTA was significantly reduced (Fig. 6A,B, ***P < 0.001, one-way ANOVA with Tukey’s multiple-comparisons test). Consistently, fiber photometry recordings revealed a significant decrease in Ca²⁺ activity in VTA glutamatergic neurons (Fig. 6C–G, **P < 0.01, two-sample Student’s *t* test). To determine whether reduced VTA activity mediates the analgesic effects of AK7 knockdown, we optogenetically activated glutamatergic neurons in the VTA (Fig. 6H). This manipulation reversed the attenuation of inflammatory hyperalgesia induced by AK7 knockdown, restoring pain sensitivity in CFA mice (Fig. 6I,J, ***P < 0.001, two-way ANOVA followed by Sidak’s multiple-comparisons test). These results indicate that suppression of VTA glutamatergic neuron activity is required for AK7 knockdown-mediated analgesia.

Conversely, overexpression of AK7 in the ACC of CON mice (Fig. 6K) significantly increased c-Fos expression in the VTA (Fig. 6L, ***P < 0.001, one-way ANOVA with Tukey’s multiple-comparisons test) and enhanced Ca²⁺ activity in VTA glutamatergic neurons (Fig. 6M–Q, **P < 0.01, ***P < 0.001, two-sample Student’s *t* test). Importantly, optogenetic inhibition of VTA glutamatergic neurons abolished the AK7 overexpression–induced hyperalgesia (Fig. 6R–T, ***P < 0.001, two-way ANOVA followed by Sidak’s multiple-comparisons test). Together, these findings demonstrate that AK7 expression in ACC glutamatergic neurons modulates inflammatory pain by regulating downstream glutamatergic activity in the VTA via the ACC-VTA glutamatergic circuit, thereby establishing AK7 as a critical molecular regulator of central pain processing.

## Discussion

Chronic inflammatory pain profoundly impairs patients’ daily functioning and mental well-being. However, incomplete understanding of its central mechanisms has limited the development of effective therapeutic strategies. In the present study, we demonstrate that glutamatergic neurons within the ACC–VTA circuit play a critical role in mediating inflammatory pain in a CFA-induced mouse model. Importantly, we identify AK7 as a key molecular regulator within ACC glutamatergic neurons that modulates neuronal excitability and behavioral pain responses. These findings provide new insights into the neural circuitry and molecular mechanisms underlying inflammatory pain and suggest potential targets for therapeutic intervention.

The ACC is a pivotal limbic cortical region located within the medial prefrontal cortex and is composed predominantly of glutamatergic pyramidal neurons interspersed with a smaller population of GABAergic interneurons. The ACC integrates nociceptive inputs from ascending pathways, including the spinal cord and thalamus, and contributes to the affective–motivational dimensions of pain perception (Bonnekoh et al, 2023; Jiang et al, 2025; Wang et al, 2024). Accumulating evidence indicates that inflammatory pain induces maladaptive plasticity and functional alterations within central pain-related circuits, particularly involving glutamatergic neurotransmission (Li et al, 2021; Matisz and Gruber, 2022). Consistent with these observations, our data demonstrate robust activation of ACC glutamatergic neurons during CFA-induced inflammatory pain. The ACC sends direct projections to the VTA, forming a critical cortico-mesolimbic circuit that functionally links pain processing with motivational and affective states. Upon exposure to noxious stimuli, ACC glutamatergic neurons become activated and transmit integrated pain-related information to the VTA, thereby modulating the activity of both dopaminergic and GABAergic neurons within this region. This ACC–VTA pathway has been implicated in pain modulation and is thought to shape the affective and motivational dimensions of pain through reward–pain interactions (Song et al, 2024). Notably, our findings further highlight the crucial role of VTA glutamatergic neurons in chronic inflammatory pain. Specifically, we demonstrated that bidirectional manipulation of ACC glutamatergic neuronal activity directly altered both the activity of VTA glutamatergic neurons and pain-related behaviors. These results provide additional evidence supporting the functional importance of the ACC-VTA circuit in the regulation of chronic inflammatory pain.

However, although our anterograde viral tracing and immunofluorescence colocalization analyses revealed that ACC glutamatergic neurons predominantly innervate VTA glutamatergic neurons, the potential contribution of VTA dopaminergic neurons to the modulation of chronic inflammatory pain cannot be completely excluded. First, our immunostaining data showed that more than 10% of ACC-projecting neurons within the VTA were dopaminergic, suggesting a potential role for this neuronal population. Second, although our anatomical evidence identified VTA glutamatergic neurons as the primary postsynaptic targets of ACC projections, we cannot rule out the possibility that ACC inputs may indirectly influence VTA dopaminergic neuronal activity through local GABAergic interneurons. Future studies incorporating complementary approaches, such as dopamine sensor and in vivo microdialysis, will be necessary to further elucidate the functional organization of this pathway. In particular, these techniques may help determine whether ACC projections establish direct monosynaptic connections with VTA glutamatergic neurons or simultaneously shape VTA network activity through local inhibitory microcircuits. Such investigations will provide a more comprehensive understanding of the dynamic role of the ACC–VTA circuit in the development and maintenance of chronic inflammatory pain.

A major finding of this study is the identification of AK7 as a molecular mediator of inflammatory pain within ACC glutamatergic neurons. AK7 is a member of the adenylate kinase family, enzymes that catalyze the reversible interconversion of adenine nucleotides (ATP, ADP, and AMP), thereby maintaining cellular energy homeostasis and influencing energy-dependent signaling processes (Ionescu, 2019; Orädd et al, 2021; Pääsuke et al, 2016; Song and Zhu, 2013). Although the role of AK7 in pain regulation has been largely unexplored, kinases and energy metabolism–related enzymes have increasingly been recognized as important modulators of neuronal excitability and synaptic plasticity in pain states (Liu et al, 2021; Zhai et al, 2023; Zhang et al, 2020). We found that AK7 expression was significantly upregulated in ACC glutamatergic neurons following CFA-induced inflammation. Selective knockdown of AK7 in these neurons alleviated inflammatory hyperalgesia, reduced ACC neuronal activation, and suppressed downstream VTA activity. Conversely, AK7 overexpression in ACC glutamatergic neurons of control mice was sufficient to induce pain hypersensitivity and enhance VTA neuronal activity. These findings indicate that AK7 plays a pro-nociceptive role within the ACC-VTA circuit. Mechanistically, AK7 may facilitate inflammatory pain by enhancing the excitability of ACC glutamatergic neurons. Given its role in adenine nucleotide metabolism, AK7 may influence neuronal energy availability, ATP-dependent ion channel function, or synaptic transmission efficiency, thereby promoting glutamatergic output from the ACC. Enhanced excitatory drive from the ACC to the VTA could amplify pain-related signaling and contribute to the persistence of inflammatory pain. Although the precise downstream molecular pathways remain to be elucidated, it is plausible that AK7 regulates synaptic plasticity, neurotransmitter release, or intracellular signaling cascades within ACC neurons that strengthen ACC-VTA connectivity during chronic pain.

Within the broader context of existing literature, our study extends current understanding of pain-related neural circuits by integrating molecular, cellular, and circuit-level mechanisms. While previous studies have emphasized the role of the ACC in pain processing, the functional contribution of the VTA and the involvement of AK7 within the ACC-VTA axis have remained largely unexplored. Our findings bridge this gap and provide a more comprehensive framework for understanding how cortical metabolic regulators influence mesolimbic pain circuits. Importantly, the identification of AK7 as a key regulator within the ACC-VTA circuit highlights a novel therapeutic target for inflammatory pain. Modulating AK7 expression or activity may offer a targeted approach to attenuate central sensitization while potentially avoiding the limitations and adverse effects associated with current analgesic therapies. Despite these mechanistic insights, several limitations should be acknowledged. In the present study, our assessment of metabolic alterations was primarily focused on key indicators of cellular energy homeostasis, including the AMPK signaling pathway, ATP/ADP ratio, and adenosine levels. However, mitochondrial function was not directly evaluated, and the AK7-dependent metabolic regulatory pathway was not further validated through pharmacological or genetic manipulation in primary cultured neurons. Future studies integrating mitochondrial functional analyses with in vitro metabolic interventions will help clarify how AK7 regulates neuronal energy metabolism and define the upstream regulatory mechanisms and downstream signaling pathways underlying its effects on neuronal excitability. These investigations will provide a stronger mechanistic basis for evaluating AK7 as a potential therapeutic target for chronic pain. In summary, this study demonstrates that upregulation of AK7 enhances the excitability of ACC glutamatergic neurons and promotes chronic inflammatory pain through the ACC-VTA circuit. These findings advance our understanding of central sensitization mechanisms in inflammatory pain and identify AK7 as a promising molecular target for the development of novel pain therapies.

## Methods

**Table:** Reagents and tools table

Reagent/resource	Reference or source	Identifier or catalog number
**Experimental models**		
C57BL/6 mice (6–12 weeks old)	Hangzhou Qizhen Experimental Animal Technology Co., Ltd	ARD-MOUSE
**Recombinant DNA**		
**RT-qPCR primers**		
Mouse AK7	Forward: TGAGACGGTCTTCAACTACTTC	
	Reverse: GAGCTGTTTGATAGCAAGTCTG	
Mouse-GAPDH	Forward: GGTTGTCTCCTGCGACTTCA	
	Reverse: TGGTCCAGGGTTTCTTACTCC	
**Antibodies**		
Anti-glutamate	Sigma-Aldrich	G6642
Anti-GAD67	Abcam	ab213508
Anti-c-Fos	OASIS TECH	0B-PGP080-02
Anti-TH	Proteintech	25859-1-AP
Anti-AK7	Thermo Fisher Scientific	PA5-109375
Anti-p-AMPK	Proteintech	86794-1-RR
Anti-β-actin	Proteintech	66009-1-Ig
**Chemicals, enzymes and other reagents**		
Complete Freund’s adjuvant	sigma	F5881
Adenosine Colorimetric Assay Kit	Elabscience	E-BC-K833-M
ATP/ADP Ratio Chemiluminescence Assay Kit	Elabscience	E-BC-F004
AAV2/9-vglut2-GCaMP6f	BrainVTA	PT-3722
AAV2/9-vglut2-eNpHR-EYFP	BrainVTA	PT-3725
AAV2/9-vglut2-ChR2-EYFP	BrainVTA	PT-3724
AAV2/9-vglut2-hM4Di-EYFP	BrainVTA	PT-3883
AAV2/9-vglut2-hM3Dq-EYFP	BrainVTA	PT-8573
AAV2/9-hSyn-EGFP	BrainVTA	PT-1990-9
AAV2/R-hSyn-EGFP	BrainVTA	PT-1990-R
AAV2/1-Vglut2-Cre	BrainVTA	PT-2592
AAV2/9-EF1α-DIO-EYFP	BrainVTA	PT-0795
rAAV-vglut2-AK7-P2A-mCherry-WPRE-hGH	BrainVTA	PT-20500
rAAV-vglut2-mCherry-5’miR-30a-shRNA(AK7)-3’miR-30a-WPREs	BrainVTA	PT-20501
**Software**		
GraphPad Software	GraphPad Software Inc	8.0.0 / 8.4.3
**Other**		
Stereotaxic apparatus	RWD	71,000-M
Infusion pump	Longer Pump	TJ-2A
Fiber photometry system	RWD	R821
Optogenetics system	RWD	IOS-465/589/635
Optical fiber	RWD	R-FOC-BL200C-39NA

### Animals

Animals were housed in accordance with standardized laboratory animal care guidelines, with a maximum of five mice per cage. All mice were maintained under controlled environmental conditions (temperature: 23 ± 1 °C; humidity: 40–60%) with a 12-h light/dark cycle and had ad libitum access to food and water. All experimental procedures were approved by the Ethics Committee of Zhangjiagang First People’s Hospital (approval No: ZJGYYLL-2025-04-022) and the behavioral tests were in accordance with the requirements of the International Association for the Study of Pain.

### Induction of inflammatory pain

Complete Freund’s adjuvant (CFA) is widely used to establish experimental models of inflammatory pain (Larson et al, 1986). CFA induces localized inflammation and pain sensitization through subcutaneous injection. In this study, 10 μL of CFA was injected into the plantar surface of the hind paw to induce a robust local inflammatory response. Control (CON) mice received an equivalent volume of saline solution.

### Behavioral testing

Mechanical sensitivity was assessed using von Frey monofilaments (Gu et al, 2020). Mice were acclimated in individual Plexiglas chambers for 1 h prior to testing. A series of von Frey filaments (0.008, 0.02, 0.04, 0.07, 0.16, 0.4, 0.6, 1, 1.4, 2, and 4 g) was applied to the plantar surface of the hind paw to evaluate mechanical withdrawal responses. Each stimulus was applied five times at 1-min intervals. A positive response was defined as toe spreading, rapid paw withdrawal, or paw licking.

Thermal hyperalgesia was measured using an analgesia meter (Hargreaves et al, 1988). Mice were acclimated in Plexiglas chambers for 1 h. When the mouse was stationary, a focused radiant heat source was applied to the plantar surface of the hind paw. Paw withdrawal latency was recorded as the time required for paw retraction. Measurements were repeated five times per paw at 5-min intervals, and the average value was used for analysis.

### Immunofluorescence staining

Mice were perfused intracardially with phosphate-buffered saline (PBS), followed by 4% paraformaldehyde. Brains were post-fixed overnight at 4 °C and sequentially cryoprotected in 10%, 20%, and 30% sucrose solutions. Coronal brain sections (30 μm) were prepared using a Leica CM1950 cryostat. Sections were brought to room temperature and subjected to antigen retrieval twice in boiling sodium citrate buffer for 10 min each. After washing three times with PBS (10 min per wash), sections were blocked for 1 h in PBS containing 7% donkey serum and 0.3% Triton X-100. Sections were then incubated overnight at 4 °C with primary antibodies. Following three PBS washes, sections were incubated with appropriate secondary antibodies at room temperature for 1 h, washed again, and mounted with DAPI-containing mounting medium. The Primary antibodies included rabbit anti-glutamate (1:200), rabbit anti-GAD67 (1:200), pig anti-c-Fos (1:200;), rabbit anti-TH (1:200) and rabbit anti-AK7 (1:200;).

### Stereotaxic injection

Male mice aged 6–8 weeks were anesthetized with continuous isoflurane and secured in a stereotaxic apparatus. After iodophor disinfection, a midline incision was made to expose the skull. Stereotaxic coordinates were determined for the ACC (AP: +1.0 mm, ML: +0.3 mm, DV: −1.8 mm) and the VTA (AP: −3.28 mm, ML: +0.1 mm, DV: −4.2 mm), the reference point for the above coordinate dissection is the bregma. A small craniotomy was performed, and viral vectors were injected at a rate of 20 nL/min for 10 min using an infusion pump, delivering a total volume of 200 nL. The injection needle was left in place for an additional 10 min to prevent reflux.

### Fiber photometry system

Mice received unilateral injections of AAV2/9-vglut2-GCaMP6f into the right ACC or VTA. Following viral injection, an optical fiber cannula was implanted at the same site. Apply a 1.0 g von Frey filament to the plantar surface of the injected hind paw for a duration of 1–2 s. Allow an inter-stimulus interval of at least 60 s between consecutive applications. Calcium signals were recorded using a fiber photometry system equipped with 470 nm and 405 nm excitation channels, with the 405 nm channel serving as an isosbestic reference to minimize motion artifacts and signal interference (Zheng et al, 2024). Changes in GCaMP6f fluorescence (ΔF/F₀) were analyzed to reflect Ca²⁺ activity in glutamatergic ACC/VTA neurons in response to von Frey stimulation.

### Optogenetic manipulation

For optogenetic inhibition, AAV2/9-vglut2-eNpHR-EYFP was injected unilaterally into the ACC or VTA of CFA-treated mice. For optogenetic activation, AAV2/9-vglut2-ChR2-EYFP was injected into CON mice. Optical fibers were implanted following viral injection. Behavioral testing was performed during light stimulation using a STSI Optogenetics LED system, delivering either continuous yellow light (594 nm, 3–5 mW) or pulsed blue light (473 nm, 2–5 mW, 20-ms pulses at 10 Hz) for 20 s.

### Chemogenetic manipulation

To assess the regulatory role of the ACC-VTA glutamatergic circuit in inflammatory pain, a dual-virus strategy was employed. AAV2/9-vglut2-hChR2-EYFP or AAV2/9-vglut2-eNpHR-EYFP was injected into the ACC, and ceramic ferrules were implanted in the VTA to enable optical modulation of ACC-derived axon terminals. Concurrently, AAV2/9-vglut2-hM4Di-EYFP or AAV2/9-vglut2-hM3Dq-EYFP was injected into the VTA to chemogenetically inhibit or activate glutamatergic neurons. Three weeks after viral expression, optogenetic and chemogenetic manipulations were performed, and changes in inflammatory pain behaviors were assessed.

### AK7 knockdown and overexpression

To investigate the role of AK7 in CFA-induced pain, rAAV-vglut2-mCherry-5′miR-30a-shRNA (AK7)-3′miR-30a-WPREs was injected into CFA-treated mice, while rAAV-vglut2-AK7-P2A-mCherry-WPRE-hGHpA was injected into control mice. AK7 expression in the ACC was verified by Western blotting 21 days post-injection. For *AK7* knockdown, the short-hairpin sequence 5’-TAGTGAAGCCACAGA TGTA-3’ was used.

### Transcriptome sequencing

Transcriptome profiling was performed using mice injected with saline (*n* = 5) or CFA (*n* = 5). On day 3 post-injection, mice were sacrificed and right ACC tissues were collected for RNA sequencing (RNA-seq) analysis. Total RNA was extracted, purified, and processed for reverse transcription, library preparation, and sequencing according to the manufacturer’s protocols (Illumina, San Diego, CA, USA). RNA-seq libraries were constructed using the Illumina® Stranded mRNA Prep, Ligation Kit (Illumina, San Diego, CA, USA) with 1 μg of total RNA per sample. Briefly, messenger RNA was enriched by poly (A) selection using oligo (dT) magnetic beads and subsequently fragmented using fragmentation buffer. First- and second-strand complementary DNA (cDNA) were synthesized using random hexamer primers. The resulting double-stranded cDNA underwent end repair, phosphorylation, and adaptor ligation following the standard library construction protocol. Libraries were size-selected using magnetic beads to obtain cDNA fragments of ~300–400 bp and then amplified by PCR for 10–15 cycles. Library concentration was quantified using a Qubit 4.0 fluorometer, and sequencing was performed using paired-end 150 bp reads (PE150) on either the NovaSeq X Plus platform with the NovaSeq Reagent Kit (Illumina) or the DNBSEQ platform using the DNBSEQ Reagent Kit (FCL PE150, version 3.0). Differential gene expression analysis between the saline and CFA groups was conducted using DESeq2 based on normalized expression values.

### Real‑time quantitative PCR (RT-qPCR)

AK7 mRNA expression in the ACC of control and CFA-treated mice was quantified using RT-qPCR. Total RNA was extracted from ACC tissue using TRIzol reagent, and RNA concentration was measured spectrophotometrically. Reverse transcription was performed using a commercial kit (Vazyme Biotech). Quantitative PCR was conducted in a 10 μL reaction volume according to the manufacturer’s protocol.

### Western blotting

ACC tissues were homogenized in lysis buffer containing protease inhibitors (Sigma) using ultrasonic disruption (three cycles with 10-min intervals). Protein samples were separated by SDS-PAGE (Jiangsu Keygen Biotech Corp., China) and transferred to nitrocellulose membranes. Membranes were blocked with 5% skim milk for 1 h, incubated with primary antibodies overnight at 4 °C, and then incubated with secondary antibodies for 1 h at room temperature. Protein bands were visualized using chemiluminescence. Primary antibodies included anti-AK7 (1:1000), anti-P-AMPK (1:2000) and anti-β-actin (1:5000).

### Whole-cell patch-clamp recording

The whole-cell patch-clamp recording technique was employed to assess the action potentials (APs) and spontaneous excitatory postsynaptic currents (sEPSCs) of neurons in the anterior cingulate cortex (ACC) of CFA mice on the third day after induction. Electrophysiological recordings were conducted using a MultiClamp 700B patch-clamp amplifier (Molecular Devices, USA) combined with a VT1200S vibrating microtome (Leica, Germany), BX51WI upright microscope (Olympus, Japan), Sutter P-1000 programmable horizontal micropipette puller (Sutter Instrument, USA), and 1550 A analog-to-digital converter. Electrophysiological signals were acquired and recorded using Clampex 10 software (Molecular Devices, USA), while offline data analysis was performed using Clampfit software (Molecular Devices, USA). Statistical analyses were conducted using GraphPad Prism software (GraphPad Software, USA). Before recording, brain slices were maintained in a continuously oxygenated artificial cerebrospinal fluid (ACSF)-perfused recording chamber. To selectively record excitatory synaptic transmission, 100 μM picrotoxin (PTX) was added to the ACSF to block GABAergic inhibitory synaptic currents. Brain slices were transferred to the recording chamber and stabilized under an upright microscope. ACC neurons were identified under bright-field illumination based on anatomical location and morphological characteristics. Patch pipettes with a resistance of 4–6 MΩ were fabricated using a micropipette puller and filled with intracellular recording solution. Whole-cell recordings were established by approaching the target neurons, forming a high-resistance gigaseal, and subsequently rupturing the membrane. Neuronal electrophysiological properties were continuously monitored, and recordings were initiated after stabilization of the whole-cell configuration. APs and sEPSCs were recorded using predefined acquisition protocols, with each neuron recorded continuously for 5 min. Only neurons exhibiting stable baseline electrophysiological properties were included for subsequent analysis.

### Measurement of adenosine and ATP/ADP levels

The levels of adenosine and ATP/ADP ratios in the ACC tissue of mice were measured using an Adenosine Colorimetric Assay Kit and an ATP/ADP Ratio Chemiluminescence Assay Kit, respectively. All procedures were performed according to the manufacturer’s instructions.

### Statistical analysis

Data are presented as mean ± SEM. Statistical analysis was performed using Prism 8.0 software. The data in the research were assessed by one-way ANOVA, two-way ANOVA and two-sample Student’s *t* test. *P* < 0.05 was considered a statistically significant difference.

## Supplementary information

Peer Review File

Expanded View Figures

## Data Availability

The transcriptome sequencing results have been uploaded to the NCBI SRA database, with the citation number PRJNA1479699. The source data of this paper are collected in the following database record: biostudies:S-BSST3196 (https://www.ebi.ac.uk/biostudies/studies/S-BSST3196). The source data of this paper are collected in the following database record: biostudies:S-SCDT-10_1038-S44319-026-00940-z.
