# Supplementary material for: Upregulation of AK7 in ACC-VTA glutamatergic projections contributes to chronic inflammation pain in male mice
Source: EMBO Rep. 2026 Sep 21;27(20):5860–80. doi: 10.1038/s44319-026-00940-z (PMC13694706; doi:10.1038/s44319-026-00940-z)
Supplement: Supplementary file 1 [file 44319_2026_940_MOESM1_ESM.pdf]

EMBO reports

Upregulation of AK7 in ACC-VTA glutamatergic projections contributes to chronic inflammation pain

Jie Huang, Meng-Ge Li, Ru-Yu Yan, Zhen-Hua Xu, Chen-Hao Zhang, Ying Cao, Xiao-Dong Xu, and Yong-Chang Li

Corresponding author(s): Yong-Chang Li (yongchangli@suda.edu.cn) , Yong-Chang Li (yongchangli@suda.edu.cn), Ying Cao (caoying0706@suda.edu.cn), Xiao-Dong Xu (xuxiaodong0431@suda.edu.cn)

Review Timeline:

Submission Date:	16th Jan 26
Editorial Decision:	10th Apr 26
Revision Received:	9th Jul 26
Editorial Decision:	4th Aug 26
Revision Received:	14th Aug 26
Accepted:	7th Sep 26

Editor: Esther Schnapp / Martina Rembold

Transaction Report:

(Note: With the exception of the correction of typographical or spelling errors that could be a source of ambiguity, letters and reports are not edited. Depending on transfer agreements, referee reports obtained elsewhere may or may not be included in this compilation. Referee reports are anonymous unless the Referee chooses to sign their reports.)

Dear Dr. Li,

Thank you for your patience while your manuscript was peer-reviewed at EMBO reports. We have now received the full set of referee reports as well as referee cross-comments that are all pasted below.

As you will see, all referees acknowledge that your findings are potentially interesting. However, both referees 2 and 3 also have a number of comments and clear suggestions for how the study could be improved and strengthened. Given the number of points raised, I asked both referees to please specify the most important points that can realistically be addressed within the 3-4 months revision period, and they have done so in their cross-comments. Given that both referees agree on the majority of these points, I think they should be addressed. I understand that this means a major round of revision and I am happy to discuss the exact revision requirements further, if you wish to do so. This is also possible in a video chat, if you like.

I would also understand, of course, if you rather prefer to submit your ms elsewhere, given the extensive revisions required. In this case, please send us a brief note so that we can withdraw the ms.

Should you decide to embark on the revisions, I would like to note that the referee concerns must be fully addressed and their suggestions taken on board. Please address all referee concerns in a complete point-by-point response.

Acceptance of the manuscript will depend on a positive outcome of a second round of review. It is EMBO reports policy to allow a single round of major revision only and acceptance or rejection of the manuscript will therefore depend on the completeness of your responses included in the next, final version of the manuscript.

We realize that it is difficult to revise to a specific deadline. In the interest of protecting the conceptual advance provided by the work, we recommend a revision within 3 months (11th Jul 2026). Please discuss the revision progress ahead of this time with the editor if you require more time to complete the revisions.

IMPORTANT NOTE: we perform an initial quality control of all revised manuscripts before re-review. Your manuscript will FAIL this control and the handling will be DELAYED if the following APPLIES:
1) A data availability section providing access to data deposited in public databases is missing. If you have not deposited any data, please add a sentence to the data availability section that explains that.
2) Your manuscript contains statistics and error bars based on n=2. Please use scatter blots in these cases. No statistics should be calculated if n=2.

When submitting your revised manuscript, please review the submission guidelines (https://link.springer.com/journal/44319/submission-guidelines#cms-Revised-submissions) and carefully review the instructions that follow below. Failure to include requested items will delay the evaluation of your revision.

1) a .docx formatted version of the manuscript text (including legends for main figures, EV figures and tables). Please make sure that the changes are highlighted to be clearly visible.

2) individual production quality figure files as .eps, .tif, .jpg (one file per figure). See hhttps://media.springernature.com/original/springer-cms/rest/v1/content/27825798/data/v1 for more info on how to prepare your figures.

3) We replaced Supplementary Information with Expanded View (EV) Figures and Tables that are collapsible/expandable online. A maximum of 5 EV Figures can be typeset. EV Figures should be cited as 'Figure EV1, Figure EV2" etc... in the text and their respective legends should be included in the main text after the legends of regular figures.

- For the figures that you do NOT wish to display as Expanded View figures, they should be bundled together with their legends in a single PDF file called *Appendix*, which should start with a short Table of Content. Appendix figures should be referred to in the main text as: "Appendix Figure S1, Appendix Figure S2" etc. See 'Expanded View' section of the submission guidelines.

- Additional Tables/Datasets should be labeled and referred to as Table EV1, Dataset EV1, etc. Legends have to be provided in a separate tab in case of .xls files. Alternatively, the legend can be supplied as a separate text file (README) and zipped together with the Table/Dataset file.

4) a .docx formatted letter INCLUDING the reviewers' reports and your detailed point-by-point responses to their comments. As part of the EMBO Press transparent editorial process, the point-by-point response is part of the Review Process File (RPF), which will be published alongside your paper.

5) a complete author checklist, which you can download from our author guidelines. Please insert information in the checklist that is also reflected in the manuscript. The completed author checklist will also be part of the RPF.

6) Please note that all corresponding authors are required to supply an ORCID ID for their name upon submission of a revised manuscript (<https://orcid.org/>), which can be linked to your profile in the manuscript submission system.

7) Before submitting your revision, primary datasets produced in this study need to be deposited in an appropriate public database. Please remember to provide a reviewer password if the datasets are not yet public. The accession numbers and database should be listed in a formal "Data Availability" section placed after Methods. Please note that the Data Availability Section is restricted to new primary data that are part of this study. * Note - All links should resolve to a page where the data can be accessed. *

If your study has not produced novel datasets, please mention this fact in the Data Availability Section.

8) At EMBO Press we ask authors to provide source data for the main manuscript figures. You will receive a separate email with instructions for providing source data with your revised manuscript, including how to upload and organize the files.

9) Our journal also encourages inclusion of *data citations in the reference list* to directly cite datasets that were re-used and obtained from public databases. Data citations in the article text are distinct from normal bibliographical citations and should directly link to the database records from which the data can be accessed. In the main text, data citations are formatted as follows: "Data ref: Smith et al, 2001" or "Data ref: NCBI Sequence Read Archive PRJNA342805, 2017". In the Reference list, data citations must be labeled with "[DATASET]". A data reference must provide the database name, accession number/identifiers and a resolvable link to the landing page from which the data can be accessed at the end of the reference.

10) Regarding data quantification, the following points must be specified in each figure legend:

- the name of the statistical test used to generate error bars and P values,

- the number (n) of independent experiments (please specify technical or biological replicates) underlying each data point,

- the nature of the bars and error bars (s.d., s.e.m.),

- If the data are obtained from n Program fragment delivered error ``Can't locate object method "less" via package "than" (perhaps you forgot to load "than"?) at //ejpvfs23/sites23b/embor_www/letters/embor_decision_revise_and_review.txt line 54.' 2, use scatter blots showing the individual data points.

Discussion of statistical methodology can be reported in the materials and methods section, but figure legends should contain a basic description of n, P and the test applied.

- Please also include scale bars in all microscopy images.

11) The journal requires a statement specifying whether or not authors have competing interests (defined as all potential or actual interests that could be perceived to influence the presentation or interpretation of an article). In case of competing interests, this must be specified in your disclosure statement.

12) All Materials and Methods need to be described in the main text using our 'Structured Methods' format, which is required for all research articles. According to this format, the Methods section includes a separate Reagents and Tools Table file (listing key reagents, experimental models, software and relevant equipment and including their sources and relevant identifiers) and a Methods and Protocols section describing the methods using a step-by-step protocol format. The aim is to facilitate adoption of the methodologies across labs. More information on how to adhere to this format as well as a downloadable template (.docx) for the Reagents and Tools Table can be found in our author guidelines.

We would also welcome the submission of cover suggestions, or motifs to be used by our Graphics Illustrator in designing a cover.

As part of the EMBO publication's Transparent Editorial Process, EMBO reports publishes online a Review Process File (RPF) to accompany accepted manuscripts. This File will be published in conjunction with your paper and will include the referee reports, your point-by-point response and all pertinent correspondence relating to the manuscript.

You are able to opt out of this by letting the editorial office know (emboreports@embo.org). If you do opt out, the Review Process File link will point to the following statement: "No Review Process File is available with this article, as the authors have chosen not to make the review process public in this case."

I look forward to seeing a revised form of your manuscript when it is ready.

Kind regards,
Esther

Esther Schnapp, PhD
Senior Editor
EMBO reports

Referee #1:

This manuscript presents a compelling and rigorously executed study that provides significant novel insights into the neural circuitry and molecular substrates underlying chronic inflammatory pain. The work is characterized by a sophisticated logical progression, transitioning seamlessly from behavioral phenotyping to circuit-level dissection, and culminating in the identification of AK7 as a pivotal molecular regulator. A major strength of this study lies in its methodological breadth, integrating fiber photometry, optogenetics, chemogenetics, viral tracing, and transcriptomics to provide a multi-level validation of the findings. The data are robust, the visualizations are exemplary, and the conclusions are firmly grounded in the experimental evidence. Specifically, the identification of AK7 within the ACC-VTA glutamatergic pathway represents a substantial contribution to the field, offering promising new targets for therapeutic intervention. The manuscript is well-structured and lucidly written; the following minor revisions are intended to further enhance the rigor and clarity of this high-quality report.

Minor comments:
1.The study exclusively utilizes male C57BL/6 mice. While this approach minimizes hormonal variability, it is well-established that chronic pain exhibits significant sexual dimorphism in both clinical prevalence and underlying neurobiological mechanisms. To enhance the manuscript's transparency and generalizability, the authors should briefly acknowledge this limitation. A concise discussion on the necessity of future studies to validate these circuits and molecular pathways in female cohorts would strengthen the study's impact.
2.In the "Stereotaxic Injection" subsection, the coordinates for the ACC are well-defined (AP: +1.0 mm, ML: +0.3 mm, DV: -1.8 mm). To ensure optimal reproducibility across laboratories, please explicitly state the anatomical reference point used (e.g., "relative to bregma").
3.The qualitative co-localization data in Figures 2A and 3E (e.g., the predominant overlap of c-Fos with glutamatergic markers) are highly suggestive. However, to meet the standards of statistical granularity, the authors should provide precise quantification.
4.For the quantitative analysis presented in Figures 2A, 3E, and 4I, the data would be more effectively visualized using bar charts. A revision of these panels is suggested.

Referee #2:

The manuscript by Jie Huang et al. investigates the role of AK7 in ACC glutamatergic neurons in chronic inflammatory pain using a CFA mouse model. The authors report that the upregulation of adenylate kinase 7 (AK7) in glutamatergic neurons of the anterior cingulate cortex (ACC) regulates chronic pain through the ACC-VTA circuit. The study integrated multiple techniques, including viral tracing, optogenetics, chemogenetics, fiber photometry, and transcriptome sequencing, and the overall experimental design is technically comprehensive. However, despite the breadth of approaches employed, this work exhibits clear limitations in terms of conceptual innovation, mechanistic depth, and clinical translational significance. Furthermore, I am not convinced by the strength and interpretability of the behavioral readout, particularly regarding the anxiety-like behavior outcomes, for the reasons outlined below.

Major points:
1. The study demonstrates that AK7 is upregulated in ACC glutamatergic neurons and modulates the ACC VTA circuit during chronic inflammatory pain. However, the specific molecular pathway underlying AK7 function remains unclear. As an adenylate kinase involved in nucleotide metabolism, how AK7 modulates glutamatergic neuronal excitability and synaptic transmission-whether through metabolic regulation, ATP-dependent signaling pathways, or downstream molecular cascades-remains largely unexplored. No metabolic measurements (ATP/ADP/AMP levels, mitochondrial function, or energy stress markers) are provided. Further investigation into the downstream mechanisms by which AK7 regulates neuronal excitability is strongly recommended.
2. Although changes in neuronal activity were demonstrated using calcium signaling and c Fos labeling, no electrophysiological recordings were performed throughout the study. Alterations in neuronal excitability and synaptic transmission were inferred solely from indirect indicators, lacking direct functional validation. Complementary electrophysiological experiments -such as whole cell patch clamp recordings to assess intrinsic membrane properties, firing rates, or synaptic currents-would substantially strengthen the mechanistic conclusions.
3. Synaptic plasticity represents a central molecular model in the regulation of chronic pain. It remains unclear whether AK7 influences synaptic plasticity via the adenosine signaling pathway or other metabolic mechanisms. Given the enzymatic role of AK7 in adenine nucleotide metabolism, it would be important to determine whether AK7 modulates adenosine signaling, ATP-dependent ion channel function, or long-term synaptic adaptations that contribute to pain persistence.
4. All experiments were conducted using 6-12 week old male C57BL/6 mice. Emerging evidence indicates that the occurrence,

development, and central regulatory mechanisms of chronic inflammatory pain exhibit significant sexual dimorphism. The absence of female mice limits the generalizability of the findings. Parallel experiments addressing potential sex differences are recommended.
5. This study verifies AK7 upregulation in ACC glutamatergic neurons following CFA induced inflammatory pain but does not include multiple time points to examine the dynamic relationship among AK7 expression, neuronal activity, and pain related behaviors. Without time-course analysis, it is not possible to distinguish whether AK7 contributes primarily to the initiation phase, maintenance phase, or both stages of chronic pain. Time-dependent experiments would significantly enhance the depth of the study.
6. In the central regulation of chronic inflammatory pain, activation of astrocytes and microglia modulates neuronal synaptic transmission and circuit function. Glial cells in both the ACC and VTA are known contributors to pain sensitization. However, this study focuses exclusively on neuron specific AK7 and glutamatergic circuits, without examining glial activation in the ACC/VTA in the CFA model or assessing whether glia participate in AK7 mediated circuit regulation and pain behaviors. Further experiments investigating glial involvement are recommended.
7. Although the authors show anatomical projections and functional manipulation of ACC-VTA glutamatergic neurons, several issues remain: the VTA contains dopaminergic, GABAergic, and glutamatergic neurons, the downstream targets are not well defined, no dopamine measurements are included, and it is unclear whether ACC projections synapse directly onto VTA glutamatergic neurons or act through local interneurons. Given the known complexity of VTA microcircuits, the current circuit interpretation is overly simplified.

Minor points:
1. Figure 4 presents transcriptome data but focuses exclusively on AK7. Given that 127 differentially expressed genes (84 upregulated and 43 downregulated) were identified, the analysis is overly limited. A broader examination of the dataset would enhance the impact of the RNA-seq findings.
2. Figure 3A-C illustrates the glutamatergic projections of ACC-VTA, but lacks: precise sub-regional localization of the ACC injection site (such as ACAd vs ACAv), detailed spatial distribution of axonal terminals within the VTA (in relation to dopaminergic neuron organization) , and quantitative statistics of retrogradely labeled neurons.
3. Introduction (Section 3): In the paragraph beginning with "Adenylate kinase 7 (AK7) is a phosphotransferase..." , the description of AK7's role in the nervous system lacks direct literature support. Appropriate references should be added.
4. Line 327: The word "one" should be capitalized.

Referee #3:

This study investigates the role of AK7 in ACC glutamatergic projections to the VTA in chronic inflammatory pain, and the research topic is of potential significance for elucidating the central molecular and circuit mechanisms of chronic inflammation pain. The experimental design combines multiple technical approaches including behavioral tests, fiber photometry, optogenetics and chemogenetics, which is relatively systematic. However, the manuscript has several critical issues regarding experimental methods, data reliability, reference accuracy and mechanism elaboration that need to be thoroughly addressed and revised. The major comments are as follows:
1. The anti-GABA antibody is not a specific molecular marker for GABAergic neurons (Fig.2A, Fig.3E). Therefore, the co-localization analysis of GABA and c-Fos cannot accurately reflect the number of c-Fos-positive GABAergic neurons in the ACC and VTA. The authors are advised to use specific markers for GABAergic neurons (e.g., GAD65 or GAD67) to perform the co-localization experiment again and re-analyze the relevant data.
2. The reference cited in lines 130-132 (Does rejection hurt? An FMRI study of social exclusion) primarily focuses on the effect of social stress on negative emotions, with no relevance to somatic pain research. This citation is inconsistent with the authors' description in this section. The authors should replace this inappropriate reference with relevant literature related to somatic pain and carefully check and revise all references throughout the manuscript to ensure the consistency between the citation content and the research context.
3. In the fiber photometry experiments, the specific gram value of von Frey filament stimulation applied to induce calcium signal changes in ACC and VTA glutamatergic neurons is not clearly stated in the Materials and Methods section or the figure legends. This key experimental parameter is essential for the reproducibility of the study, and the authors need to supplement this information in detail.
4. The lower parts of the fiber photometry traces in Figure 5G (CFA+AK7-KD group), Figure 5Q (CON-NC-OE group), Figure 6D (CFA+AK7-KD group), Figure 6N (CON-NC-OE group) exhibit an unrealistic state of complete silence with no detectable calcium signal fluctuations. The authors are required to provide a detailed and reasonable explanation for this abnormal data phenomenon.
5. The optogenetic manipulation experiments lack critical validation of virus efficacy and specificity. Specifically, there is no fluorescent validation data to confirm the specific expression of optogenetic proteins (eNpHR/ChR2) in glutamatergic neurons; in addition, electrophysiological validation (e.g., patch-clamp recording in brain slices) is absent to verify whether the optogenetic stimulation can effectively activate or inhibit the activity of target neurons. The authors need to supplement these two key validation experiments and provide the corresponding experimental results.
6. The study confirms that AK7 upregulation in ACC glutamatergic neurons modulates neuronal calcium activity and pain-related behaviors, but the direct molecular mechanism by which AK7 regulates neuronal activity remains largely unclear. As an

adenylate kinase involved in nucleotide metabolism, whether AK7 upregulation alters the levels of ATP/ADP/AMP in the ACC region needs to be experimentally verified. Furthermore, the authors should further investigate whether AK7 affects glutamatergic synaptic transmission or ion channel function by regulating cellular energy metabolism, and supplement relevant in vitro and in vivo experimental evidence to elucidate the specific molecular cascade of AK7 in pain modulation.

Cross-comments from referee 2:

I have carefully read the reports from Referees 1 and 3. While I appreciate Referee 1's positive outlook on the methodological breadth, I remain convinced that the limitations identified in my initial report-and echoed in part by Referee 3-are fundamental. In response to your specific question regarding which experiments are essential and feasible within a reasonable timeframe, I would like to reiterate my stance on the following points:

1. Functional Validation via Electrophysiology (Crucial)
Referee 1 characterizes the study as "rigorous", but as Referee 3 and I both noted, the study lacks any direct measurement of neuronal function. For a high-impact journal such as EMBO Reports, relying exclusively on calcium imaging (fiber photometry) and c-Fos staining-without patch-clamp recording-to draw conclusions about changes in neuronal excitability represents a significant methodological gap. Whole-cell recordings in acute slices are the standard for verifying intrinsic excitability and synaptic currents (EPSCs/IPSCs) in the ACC-VTA circuit, which can be completed within 2-3 months.

2. The Molecular Mechanism of AK7
The core of this manuscript is the identification of AK7. However, as it stands, the link between AK7 (a nucleotide metabolism enzyme) and neuronal firing is a "black box". Simply showing that KD/OE of AK7 changes behavior does not explain how it does so. Does it shift the ATP/ADP ratio? Does it affect adenosine signaling? Measuring local ATP/ADP levels or using pharmacological blockers of adenosine receptors/K-ATP channels would begin to bridge this gap. Without exploring the metabolic-to-electrophysiological coupling, the study remains a phenomenological observation of a new gene in an old circuit.

3. VTA Circuit Complexity and Direct Targets
The current interpretation of the ACC-VTA projection is overly simplified. As I noted, the VTA contains different types of neuron. The authors should, at a minimum, provide better histological characterization of which cell types in the VTA are being targeted (e.g., co-labeling with TH for DA neurons or GAD for GABA neurons). This is a matter of re-analyzing existing tissue or performing limited new staining, which is highly feasible.

4. Sex Dimorphism (Generalizability)
I am pleased to see that both Referees 1 and 3 also flagged the exclusive use of male mice. While performing the entire study in females might be a "major" addition, adding a key behavioral validation (e.g., the AK7-KD effect on pain) in a female cohort is essential to ensure the findings aren't a sex-specific artifact.

Cross-comments from referee 3:

I have carefully read the comments from Referees 1 and 3. I am pleased to find that we share several core concerns, particularly regarding the depth of mechanistic insight and the rigor of the functional validation. Based on the collective feedback, I would like to emphasize the following points to help the authors prioritize their revisions.

1. Necessity of electrophysiological validation. I strongly agree with Referee 2 that relying solely on fiber photometry and c-Fos is insufficient for a study of this caliber. To rigorously support the conclusions regarding ACC-VTA circuit excitability and plasticity, whole-cell patch-clamp recordings in acute slices are essential. This is the only way to definitively characterize the intrinsic excitability and synaptic changes (EPSCs/IPSCs) that underpin the observed behavioral phenotypes.

2. Bridging the metabolic-to-electrophysiological gap. The current molecular evidence for AK7 is indeed thin. While we should not burden the authors with an infinite list of experiments, the "black box" between AK7 (nucleotide metabolism) and neuronal firing must be addressed. I support the suggestion to investigate whether AK7 modulates the ATP/ADP ratio or adenosine signaling. Crucially, any changes in these metabolic markers should be linked back to neuronal function via pharmacological blockers (e.g., K-ATP channel or adenosine receptor antagonists) coupled with electrophysiological readouts.

3. Temporal dynamics of AK7 expression. It is important for the authors to characterize the expression profile of AK7 across different phases of pain (induction vs. maintenance). Given the complexity of brain function, a molecule's role may shift from being a trigger in the acute phase to a driver of chronic states. Clarifying this temporal element is vital for understanding the gene's true contribution to the pathology.

4. Cellular and sub-neuronal identity. To move beyond phenomenological observations, the authors must clarify which cell types

express AK7. I recommend performing co-labeling experiments to distinguish between neurons and non-neuronal cells, followed by a finer characterization of neuronal subtypes (e.g., DA vs. GABAergic neurons in the VTA). This is highly feasible and essential for circuit specificity.

5. Addressing sex dimorphism. Aligning with the concerns of all reviewers, the exclusive use of male mice is a limitation. At a minimum, the authors should analyze the expression levels of their core molecular targets in a female cohort at two critical time points (acute and chronic phases). If a full behavioral rescue in females is too time-consuming, these expression data, accompanied by a rigorous discussion of potential sex differences, are the baseline requirement for generalizability.

In summary, while the identification of AK7 in the ACC-VTA circuit is a new finding, the manuscript requires a more mechanistic soul. By focusing on electrophysiology, metabolic coupling, and cell-type specificity, the authors may elevate this work to the standard expected by EMBO Reports.

Referee #1:

This manuscript presents a compelling and rigorously executed study that provides significant novel insights into the neural circuitry and molecular substrates underlying chronic inflammatory pain. The work is characterized by a sophisticated logical progression, transitioning seamlessly from behavioral phenotyping to circuit-level dissection, and culminating in the identification of AK7 as a pivotal molecular regulator. A major strength of this study lies in its methodological breadth, integrating fiber photometry, optogenetics, chemogenetics, viral tracing, and transcriptomics to provide a multi-level validation of the findings. The data are robust, the visualizations are exemplary, and the conclusions are firmly grounded in the experimental evidence. Specifically, the identification of AK7 within the ACC-VTA glutamatergic pathway represents a substantial contribution to the field, offering promising new targets for therapeutic intervention. The manuscript is well-structured and lucidly written; the following minor revisions are intended to further enhance the rigor and clarity of this high-quality report.

Response: Thank you very much for the positive comments and constructive suggestions.

Minor comments:

1. The study exclusively utilizes male C57BL/6 mice. While this approach minimizes hormonal variability, it is well-established that chronic pain exhibits significant sexual dimorphism in both clinical prevalence and underlying neurobiological mechanisms. To enhance the manuscript's transparency and generalizability, the authors should briefly acknowledge this limitation. A concise discussion on the necessity of future studies to validate these circuits and molecular pathways in female cohorts would strengthen the study's impact.

Response: We sincerely thank the reviewer for this valuable suggestion. We agree that considering potential sex differences is important for improving the generalizability of our findings. In response to this comment, we performed additional validation experiments in female mice, and these new data have been included as **Figure EV4** in the revised manuscript. Consistent with our observations in male mice, selective knockdown of AK7 in ACC glutamatergic neurons significantly attenuated CFA-induced mechanical and thermal hypersensitivity in female mice. These results indicate that the analgesic effect of AK7 knockdown is reproducible in both sexes and further strengthen the robustness of our conclusions. The corresponding results and figure have been incorporated into the revised manuscript.

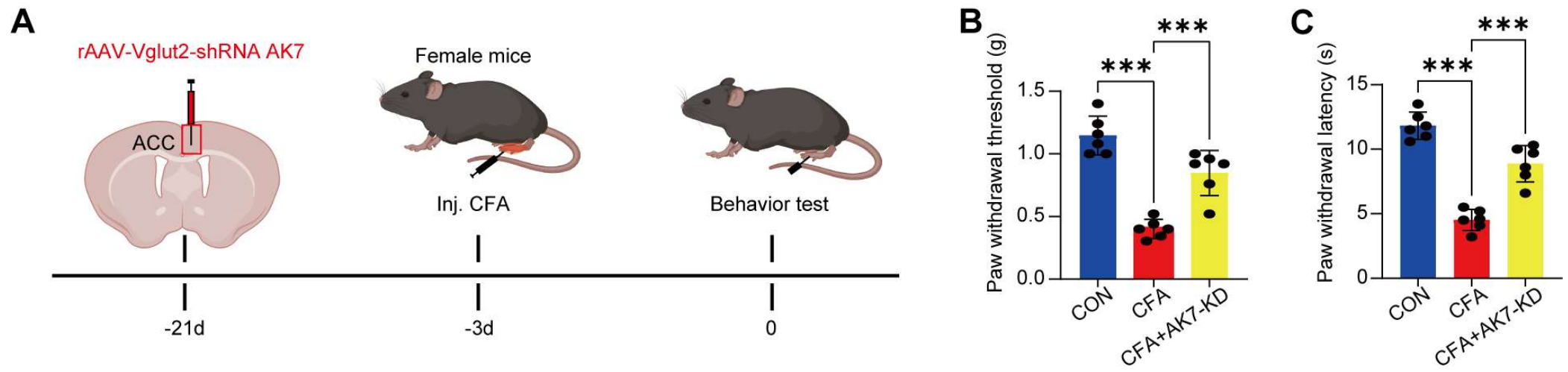

2.In the "Stereotaxic Injection" subsection, the coordinates for the ACC are well-defined (AP: +1.0 mm, ML: +0.3 mm, DV: -1.8 mm). To ensure optimal reproducibility across laboratories, please explicitly state the anatomical reference point used (e.g., "relative to bregma").

Response: As suggested, we have explicitly stated that all stereotaxic coordinates are given relative to bregma in the Materials and Methods section (Line 217, highlighted in red), thereby improving the reproducibility of our experimental procedures.

3.The qualitative co-localization data in Figures 2A and 3E (e.g., the predominant overlap of c-Fos with glutamatergic markers) are highly suggestive. However, to meet the standards of statistical granularity, the authors should provide precise quantification.

Response: Thank you for the comment. As recommended, we performed quantitative analyses of the colocalization between c-Fos-positive neurons and glutamatergic neurons in both **Figures 2A and 3E**. The corresponding quantitative data have now been incorporated into the revised figures and are presented in the revised manuscript.

4.For the quantitative analysis presented in Figures 2A, 3E, and 4I, the data would be more effectively visualized using bar charts. A revision of these panels is suggested.

Response: We appreciate the reviewer's helpful suggestion. Following this recommendation, we have replotted the quantitative data in **Figures 2A, 3E, and 4L** as bar graphs to improve data presentation and facilitate visual comparison. The revised figure panels have been updated accordingly in the revised manuscript.

Referee #2:

The manuscript by Jie Huang et al. investigates the role of AK7 in ACC glutamatergic neurons in chronic inflammatory pain using a CFA mouse model. The authors report that the upregulation of adenylate kinase 7 (AK7) in glutamatergic neurons of the anterior cingulate cortex (ACC) regulates chronic pain through the ACC-VTA circuit. The study integrated multiple techniques, including viral tracing, optogenetics, chemogenetics, fiber photometry, and transcriptome sequencing, and the overall experimental design is technically comprehensive. However, despite the breadth of approaches employed, this work exhibits clear limitations in terms of conceptual innovation, mechanistic depth, and clinical translational significance. Furthermore, I am not convinced by the strength and interpretability of the behavioral readout, particularly regarding the anxiety-like behavior outcomes, for the reasons outlined below.

Response: Thank you very much for the constructive comments.

Major points:

1. The study demonstrates that AK7 is upregulated in ACC glutamatergic neurons and modulates the ACC VTA circuit during chronic inflammatory pain. However, the specific molecular pathway underlying AK7 function remains unclear. As an adenylate kinase involved in nucleotide metabolism, how AK7 modulates glutamatergic neuronal excitability and synaptic transmission-whether through metabolic regulation, ATP-dependent signaling pathways, or downstream molecular cascades-remains largely unexplored. No metabolic measurements (ATP/ADP/AMP levels, mitochondrial function, or energy stress markers) are provided. Further investigation into the downstream mechanisms by which AK7 regulates neuronal excitability is strongly recommended.

Response: We sincerely thank the reviewer for this insightful comment. We agree that elucidating the molecular mechanism underlying AK7 function is essential for strengthening the mechanistic depth of this study. In response to this suggestion, we performed a series of additional experiments to assess key indicators of cellular energy metabolism in the ACC, and these new data have been incorporated into the revised manuscript as **Figures 5O-Q**.

Specifically, we measured the ATP/ADP ratio, adenosine levels, and the expression of phosphorylated AMPK (p-AMPK), a well-established indicator of cellular energy stress. Our results showed that CFA-induced chronic inflammatory pain was associated with a reduced ATP/ADP ratio, elevated adenosine levels, and increased p-AMPK expression in the ACC. Importantly, all of these metabolic alterations were significantly ameliorated following AK7 knockdown. Although these findings do not fully define the downstream signaling cascade of AK7, they provide new evidence that AK7 may regulate

neuronal function, at least in part, through disruption of nucleotide homeostasis and cellular energy metabolism, accompanied by activation of the AMPK-associated energy stress pathway. We have also expanded the Discussion section (Lines 732–745, highlighted in red) to further discuss this potential metabolic regulatory mechanism and its limitations.

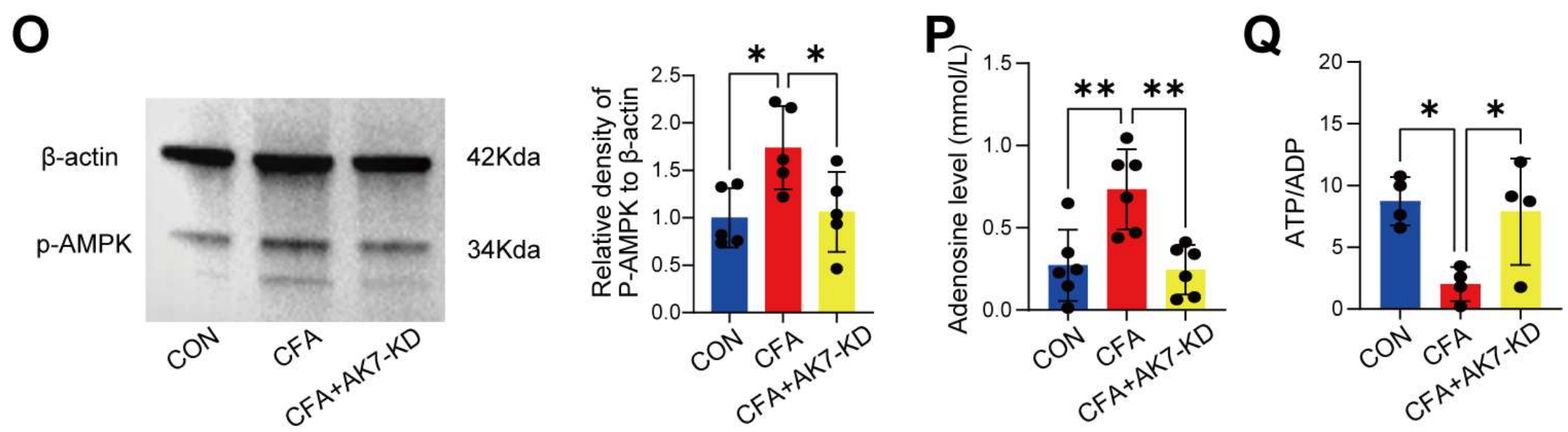

2. Although changes in neuronal activity were demonstrated using calcium signaling and c-Fos labeling, no electrophysiological recordings were performed throughout the study. Alterations in neuronal excitability and synaptic transmission were inferred solely from indirect indicators, lacking direct functional validation. Complementary electrophysiological experiments -such as whole cell patch clamp recordings to assess intrinsic membrane properties, firing rates, or synaptic currents-would substantially strengthen the mechanistic conclusions.

Response: Thank you for the comments. Accordingly, we performed ex vivo whole-cell patch-clamp recordings, and the corresponding data have been incorporated into the revised manuscript as **Figures 1J–S**. Compared with CON mice, ACC neurons from CFA mice exhibited significantly increased sEPSC frequency and amplitude, indicating enhanced excitatory synaptic transmission. In addition, CFA mice displayed increased action potential firing, depolarized resting membrane potential, and reduced rheobase current, demonstrating enhanced intrinsic neuronal excitability. These newly added electrophysiological data provide direct functional evidence that chronic inflammatory pain is accompanied by both enhanced glutamatergic synaptic transmission and increased intrinsic excitability of ACC neurons, thereby substantially strengthening the mechanistic conclusions of the study.

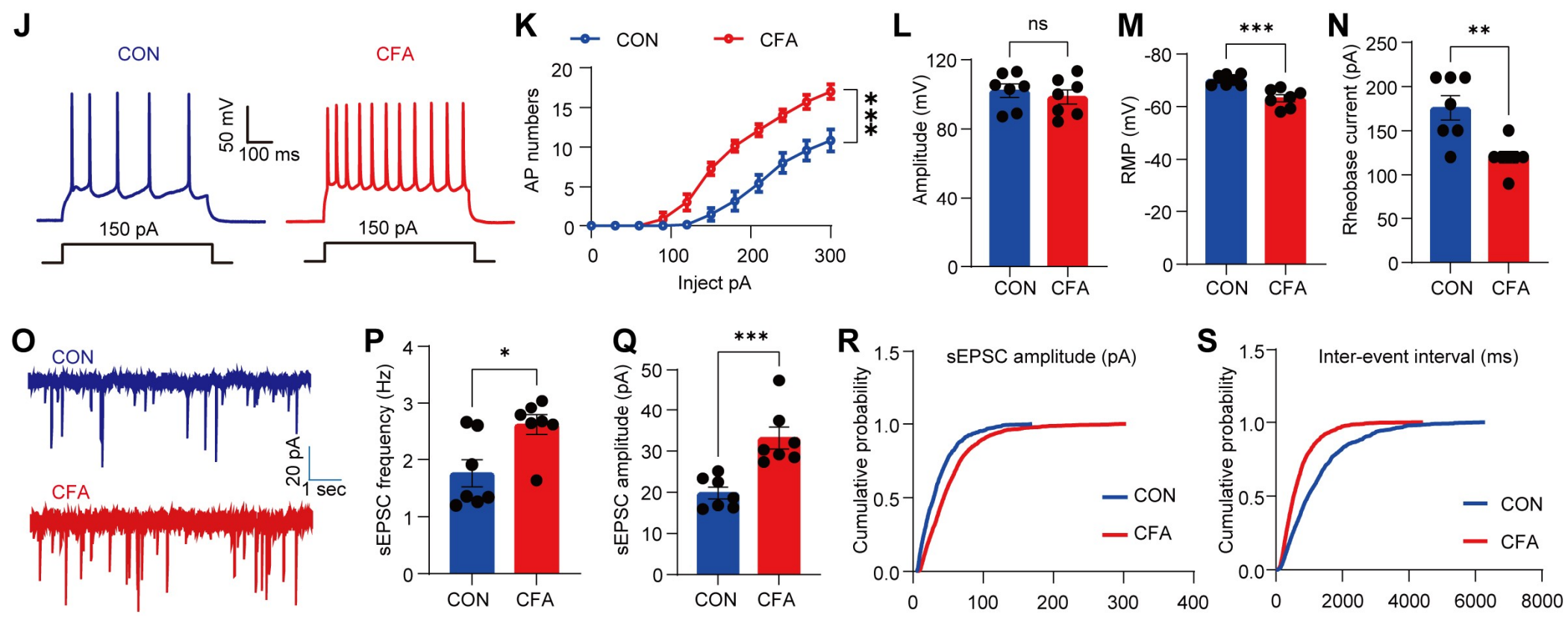

3. Synaptic plasticity represents a central molecular model in the regulation of chronic pain. It remains unclear whether AK7 influences synaptic plasticity via the adenosine signaling pathway or other metabolic mechanisms. Given the enzymatic role of AK7 in adenine nucleotide metabolism, it would be important to determine whether AK7 modulates adenosine signaling, ATP-dependent ion channel function, or long-term synaptic adaptations that contribute to pain persistence.

Response: We thank the reviewer for this insightful suggestion. We agree that clarifying whether AK7 regulates neuronal function through metabolic signaling pathways is important for understanding its contribution to chronic pain. To address this question, we supplemented whole-cell patch-clamp recordings in ACC neurons, and the corresponding electrophysiological data have been incorporated into the revised manuscript as **Figures 5D–J**. Our results demonstrated that AK7 knockdown significantly attenuated the electrophysiological abnormalities induced by CFA. Specifically, both the frequency and amplitude of sEPSCs were significantly reduced, accompanied by decreased action potential firing, hyperpolarization of the resting membrane potential, and increased rheobase current. These findings indicate that AK7 knockdown effectively suppresses enhanced glutamatergic synaptic transmission and neuronal hyperexcitability in the ACC.

In parallel, we performed additional metabolic analyses (**Figures 5O–Q**), which showed that AK7 knockdown restored the ATP/ADP ratio, reduced adenosine accumulation, and attenuated AMPK activation. Together, these findings support the notion that AK7 contributes to chronic inflammatory pain, at least in part, through disruption of cellular energy metabolism and nucleotide homeostasis. Although the precise downstream signaling mechanisms linking metabolic alterations to neuronal excitability require further investigation, the newly added data substantially strengthen the mechanistic framework of the present study.

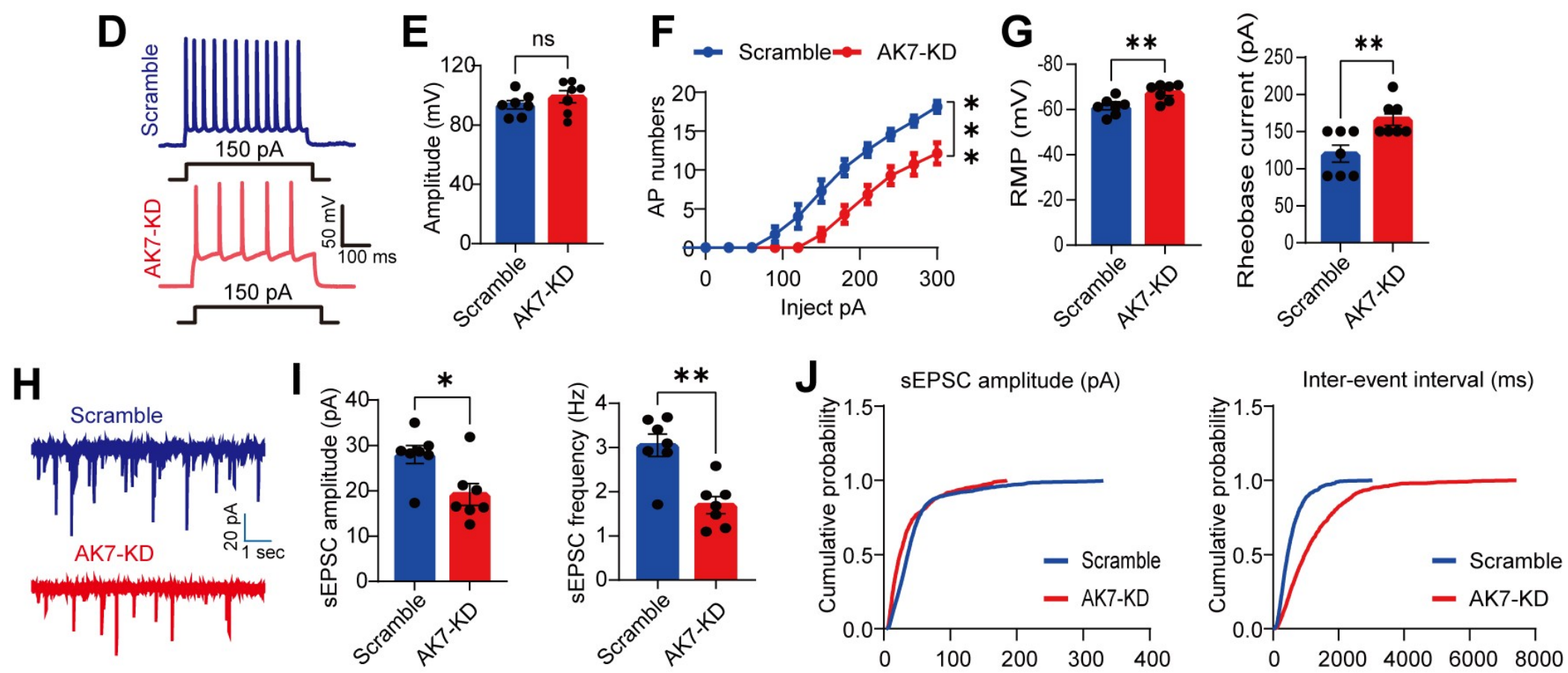

4. All experiments were conducted using 6-12 week old male C57BL/6 mice. Emerging evidence indicates that the occurrence, development, and central regulatory mechanisms of chronic inflammatory pain exhibit significant sexual dimorphism. The absence of female mice limits the generalizability of the findings. Parallel experiments addressing potential sex differences are recommended.

Response: We sincerely thank the reviewer for this valuable suggestion. We agree that considering potential sex differences is important for improving the generalizability of our findings. In response to this comment, we performed additional validation experiments in female mice, and the newly generated data have been included as **Figure EV4** in the revised manuscript. Consistent with our observations in male mice, selective knockdown of AK7 in ACC glutamatergic neurons significantly attenuated CFA-induced mechanical and thermal hypersensitivity in female mice. These results demonstrate that the analgesic effect of AK7 knockdown is reproducible in both sexes, thereby strengthening the robustness and generalizability of our findings. The corresponding results and figure have been incorporated into the revised manuscript.

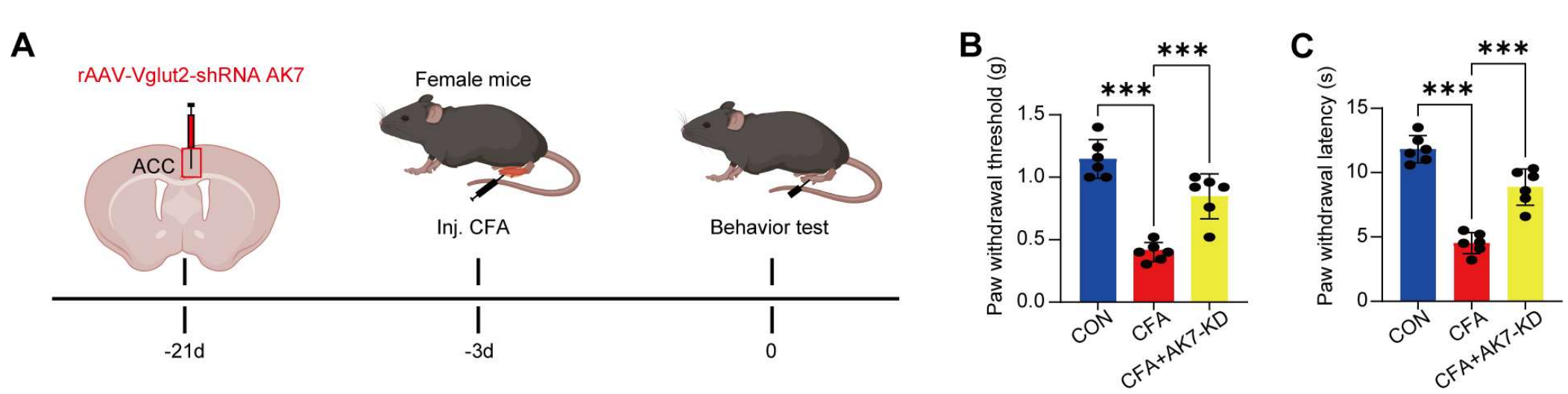

5. This study verifies AK7 upregulation in ACC glutamatergic neurons following CFA induced inflammatory pain but does not include multiple time points to examine the dynamic relationship among AK7 expression, neuronal activity,

and pain related behaviors. Without time-course analysis, it is not possible to distinguish whether AK7 contributes primarily to the initiation phase, maintenance phase, or both stages of chronic pain. Time-dependent experiments would significantly enhance the depth of the study.

Response: We thank the reviewer for this insightful suggestion. To clarify the temporal relationship between AK7 expression and the progression of chronic inflammatory pain, we performed additional Western blot analyses to examine AK7 protein expression in the ACC at multiple time points following CFA injection. As shown in the revised **Figure 4H**, AK7 expression gradually increased after CFA administration, reached its peak during the maintenance phase of chronic inflammatory pain, and remained elevated thereafter. This temporal profile closely paralleled the progression of pain-related behavioral abnormalities, suggesting that AK7 is more likely to participate in the maintenance rather than the initiation of chronic inflammatory pain. The corresponding experimental results have been added to the Results section, and the Discussion has been revised accordingly.

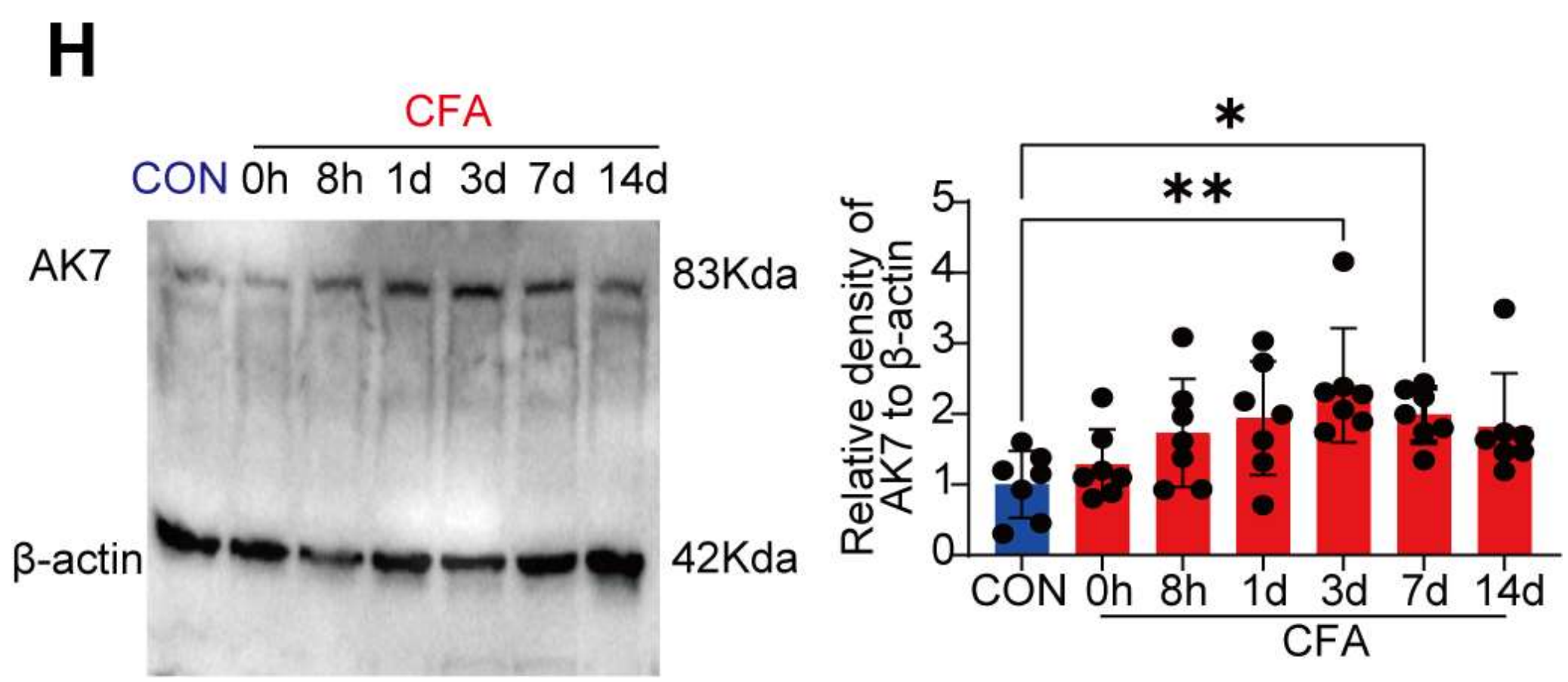

6. In the central regulation of chronic inflammatory pain, activation of astrocytes and microglia modulates neuronal synaptic transmission and circuit function. Glial cells in both the ACC and VTA are known contributors to pain sensitization. However, this study focuses exclusively on neuron specific AK7 and glutamatergic circuits, without examining glial activation in the ACC/VTA in the CFA model or assessing whether glia participate in AK7 mediated circuit regulation and pain behaviors. Further experiments investigating glial involvement are recommended.

Response: We appreciate the reviewer for raising this important point. To determine the cellular distribution of AK7 within the ACC during chronic inflammatory pain, we performed additional immunofluorescence analyses using markers for astrocytes and microglia. Our results showed that CFA treatment did not induce apparent astrocytic activation based on GFAP

immunostaining, and AK7 exhibited minimal colocalization with the microglial marker Iba1 (**Figures 4I and 4J**). These observations suggest that neuronal AK7 is likely the predominant cellular component associated with the phenotypes observed in the present study. However, we cannot completely exclude more subtle or indirect contributions of glial cells to chronic inflammatory pain, and this possibility warrants further investigation. We have incorporated these new experimental results into the revised manuscript.

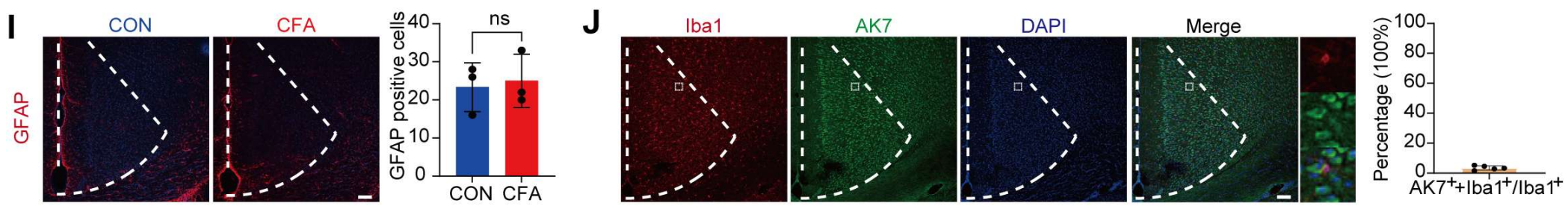

7. Although the authors show anatomical projections and functional manipulation of ACC-VTA glutamatergic neurons, several issues remain: the VTA contains dopaminergic, GABAergic, and glutamatergic neurons, the downstream targets are not well defined, no dopamine measurements are included, and it is unclear whether ACC projections synapse directly onto VTA glutamatergic neurons or act through local interneurons. Given the known complexity of VTA microcircuits, the current circuit interpretation is overly simplified.

Response: We sincerely thank the reviewer for this constructive suggestion. To further characterize the downstream neuronal populations receiving ACC glutamatergic projections, we performed additional dual-virus anterograde tracing combined with immunofluorescence colocalization analyses using markers for glutamatergic (Glutamate), GABAergic (GAD67), and dopaminergic (TH) neurons in the VTA. The results showed that ACC-derived EYFP-positive projections preferentially colocalized with Glutamate-positive neurons, whereas substantially less colocalization was observed with GAD67- or TH-positive neurons (Figure EV2). These findings suggest that VTA glutamatergic neurons are the principal postsynaptic neuronal population innervated by ACC glutamatergic projections in our experimental paradigm.

To further address the reviewer's concern, we have expanded the Discussion to acknowledge that the functional contribution of local VTA microcircuits, including indirect modulation through GABAergic interneurons and potential involvement of dopaminergic signaling, remains to be clarified by future studies employing electrophysiological recordings and high-resolution circuit-mapping approaches.

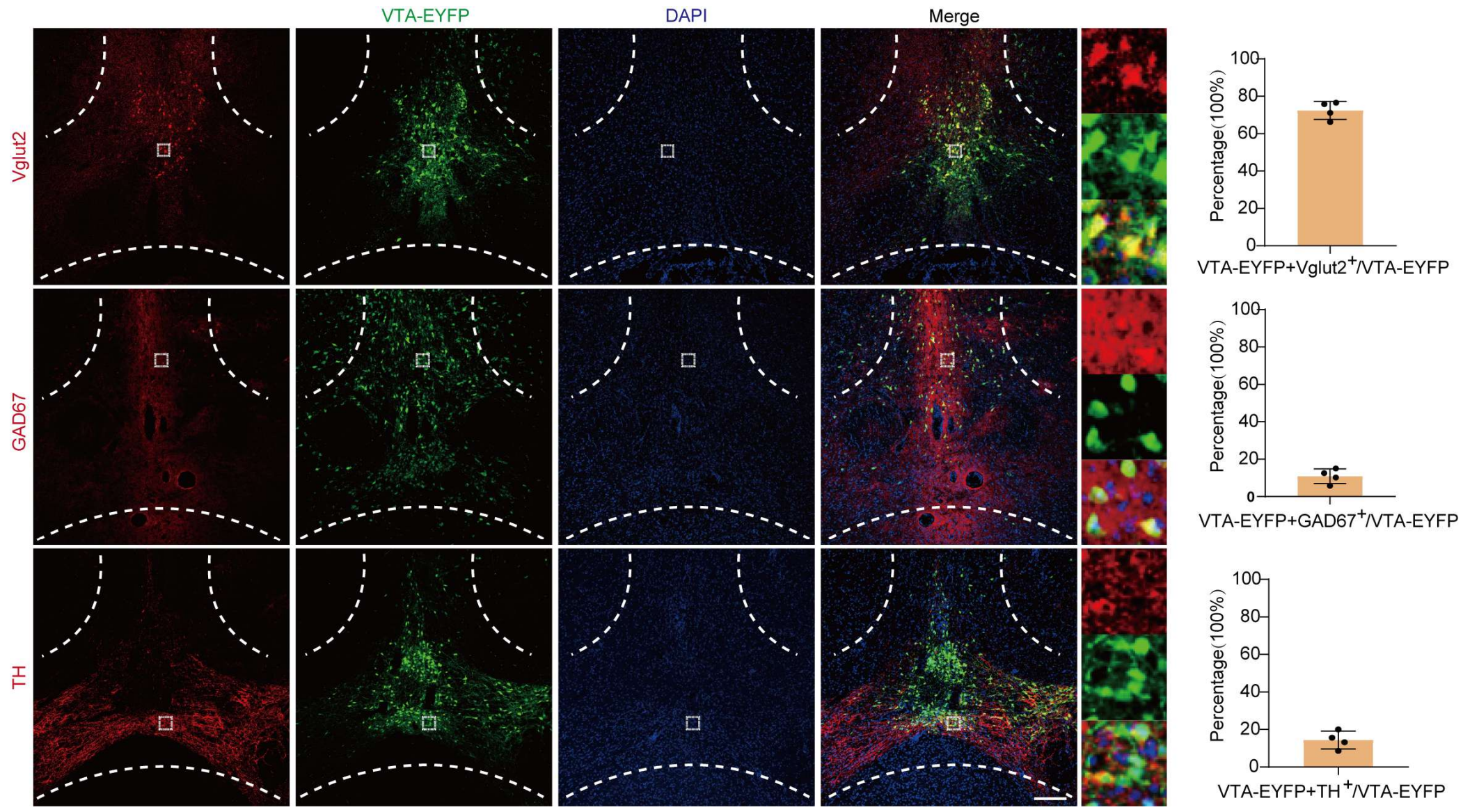

Minor points:

1. Figure 4 presents transcriptome data but focuses exclusively on AK7. Given that 127 differentially expressed genes (84 upregulated and 43 downregulated) were identified, the analysis is overly limited. A broader examination of the dataset would enhance the impact of the RNA-seq findings.

Response: We sincerely thank the reviewer for this valuable suggestion. As suggested, we performed KEGG pathway enrichment analysis and incorporated the results as **Figure 4E** in the revised manuscript. Together with the original GO enrichment analysis, these results provide a more comprehensive functional annotation of the differentially expressed genes by identifying the major biological processes and signaling pathways associated with chronic inflammatory pain. Although the present study primarily focuses on the functional characterization of AK7, the expanded GO and KEGG analyses offer a broader overview of the transcriptomic landscape and identify additional pathways that may warrant further investigation. The corresponding Results section, Figure 4E legend, and Discussion have been revised accordingly to summarize the major enriched biological processes and signaling pathways.

2. Figure 3A-C illustrates the glutamatergic projections of ACC-VTA, but lacks: precise sub-regional localization of the ACC injection site (such as ACAd vs ACAv), detailed spatial distribution of axonal terminals within the VTA (in relation to dopaminergic neuron organization), and quantitative statistics of retrogradely labeled neurons.

Response: We thank the reviewer for this constructive suggestion. In response to this comment, we have further improved the anatomical characterization of the ACC–VTA projection in the revised manuscript.

1) we have explicitly specified that the stereotaxic viral injections were targeted to the ventral ACC (vACC) and added representative histological images verifying the injection sites in **Figure 3A**.
2) we have included higher-resolution images illustrating the spatial distribution of ACC-derived axonal terminals within the VTA.
3) Quantitative analysis of retrogradely labeled ACC neurons has been performed, and the corresponding statistical results are now presented as bar graphs in the revised **Figure 3B**.

These revisions provide a more comprehensive anatomical characterization of the ACC–VTA circuit and improve the clarity and reproducibility of the anatomical data.

3. Introduction (Section 3): In the paragraph beginning with "Adenylate kinase 7 (AK7) is a phosphotransferase...", the description of AK7's role in the nervous system lacks direct literature support. Appropriate references should be added.

Response: We thank the reviewer for pointing out this omission. As suggested, we have added the appropriate references supporting the role of AK7 in the nervous system in the Introduction section of the revised manuscript (References 28 and 29, Line 142, highlighted in red).

4. Line 327: The word "one" should be capitalized.

Response: We thank the reviewer for the careful observation. The word "One" has been capitalized accordingly in the revised manuscript.

Referee #3:

This study investigates the role of AK7 in ACC glutamatergic projections to the VTA in chronic inflammatory pain, and the research topic is of potential significance for elucidating the central molecular and circuit mechanisms of chronic inflammation pain. The experimental design combines multiple technical approaches including behavioral tests, fiber photometry, optogenetics and chemogenetics, which is relatively systematic. However, the manuscript has several critical issues regarding experimental methods, data reliability, reference accuracy and mechanism elaboration that need to be thoroughly addressed and revised. The major comments are as follows:

1. The anti-GABA antibody is not a specific molecular marker for GABAergic neurons (Fig.2A, Fig.3E). Therefore, the co-localization analysis of GABA and c-Fos cannot accurately reflect the number of c-Fos-positive GABAergic neurons in the ACC and VTA. The authors are advised to use specific markers for GABAergic neurons (e.g., GAD65 or GAD67) to perform the co-localization experiment again and re-analyze the relevant data.

Response: We sincerely thank the reviewer for this valuable suggestion. We agree that GABA immunostaining is not sufficiently specific for identifying GABAergic neurons in colocalization analyses. In response to this comment, we replaced the original anti-GABA antibody with a GAD67 antibody, a well-established marker for GABAergic neurons, and repeated all immunofluorescence colocalization experiments. The updated representative images and corresponding quantitative analyses have replaced the original data in **Figures 2A and 3E** of the revised manuscript. These revisions improve the specificity and reliability of the neuronal subtype identification and further strengthen the accuracy of our conclusions.

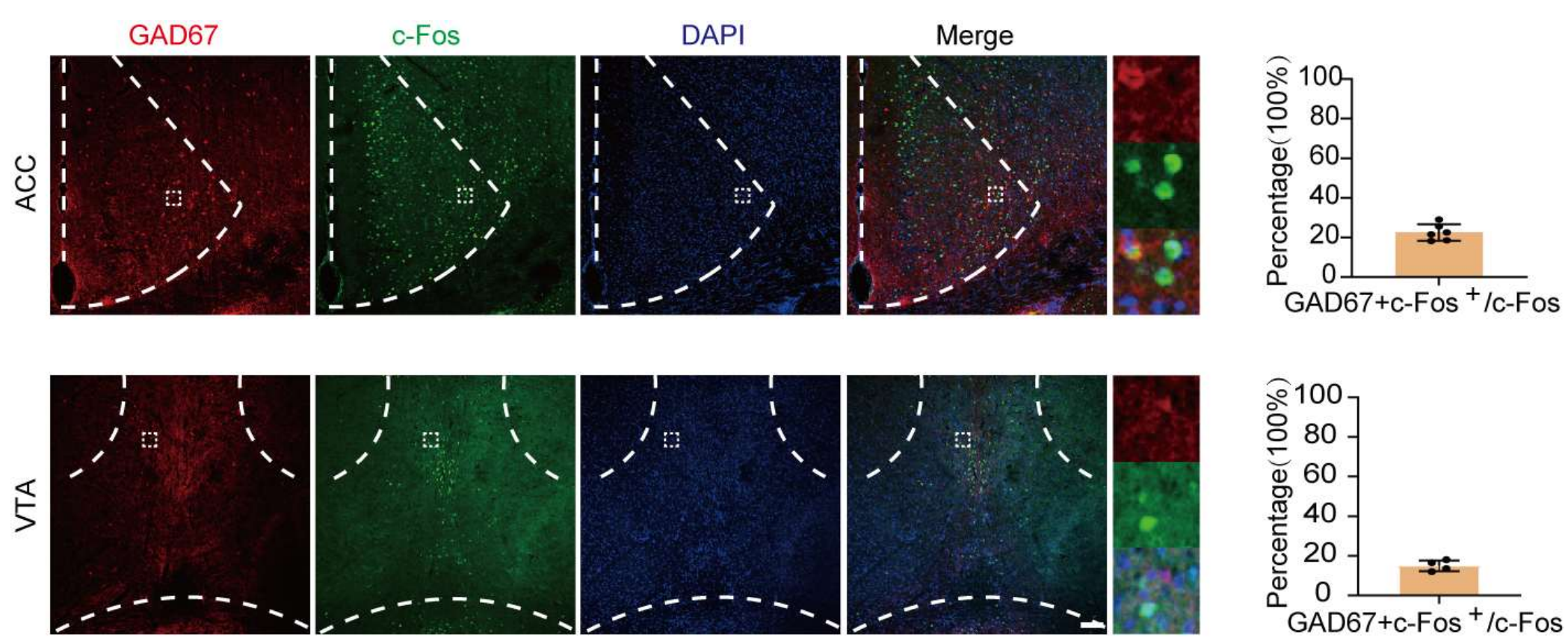

2. The reference cited in lines 130-132 (Does rejection hurt? An FMRI study of social exclusion) primarily focuses on the effect of social stress on negative emotions, with no relevance to somatic pain research. This citation is

inconsistent with the authors' description in this section. The authors should replace this inappropriate reference with relevant literature related to somatic pain and carefully check and revise all references throughout the manuscript to ensure the consistency between the citation content and the research context.

Response: We thank the reviewer for pointing out this citation error and sincerely apologize for this oversight. In response to this comment, we replaced the inappropriate reference with two relevant studies related to somatic pain (References 20 and 21, Lines 126-129, highlighted in red). In addition, we carefully reviewed all references throughout the manuscript to ensure that each citation is accurate, contextually appropriate, and consistent with the scientific statements being supported.

3. In the fiber photometry experiments, the specific gram value of von Frey filament stimulation applied to induce calcium signal changes in ACC and VTA glutamatergic neurons is not clearly stated in the Materials and Methods section or the figure legends. This key experimental parameter is essential for the reproducibility of the study, and the authors need to supplement this information in detail.

Response: We thank the reviewer for this helpful suggestion. We agree that the intensity of mechanical stimulation is an important experimental parameter for ensuring reproducibility. Accordingly, we have supplemented this information in the Materials and Methods section (Lines 227-229, highlighted in red). Specifically, a 1.0 g von Frey filament was used to evoke calcium responses during all fiber photometry recordings in both ACC and VTA glutamatergic neurons.

4. The lower parts of the fiber photometry traces in Figure 5G (CFA+AK7-KD group), Figure 5Q (CON-NC-OE group), Figure 6D (CFA+AK7-KD group), Figure 6N (CON-NC-OE group) exhibit an unrealistic state of complete silence with no detectable calcium signal fluctuations. The authors are required to provide a detailed and reasonable explanation for this abnormal data phenomenon.

Response: We sincerely thank the reviewer for carefully identifying this issue and apologize for the error in data presentation. After carefully re-examining the original datasets, we found that the apparent absence of calcium signal fluctuations in the lower portions of several heatmaps resulted from inconsistent frame-rate settings during data preprocessing prior to importing the raw recordings into the RWD analysis software. This preprocessing inconsistency affected the visualization of the heatmaps but did not alter the original calcium recordings or the quantitative analyses. To correct this issue, we standardized the frame rate for all raw fiber photometry datasets,

reprocessed the data using an identical analysis pipeline, and regenerated all corresponding heatmaps. The corrected panels (**Figures 5M, 5W, 6D, and 6N**) have replaced the original figures in the revised manuscript.

5. The optogenetic manipulation experiments lack critical validation of virus efficacy and specificity. Specifically, there is no fluorescent validation data to confirm the specific expression of optogenetic proteins (eNpHR/ChR2) in glutamatergic neurons; in addition, electrophysiological validation (e.g., patch-clamp recording in brain slices) is absent to verify whether the optogenetic stimulation can effectively activate or inhibit the activity of target neurons. The authors need to supplement these two key validation experiments and provide the corresponding experimental results.

Response: We sincerely thank the reviewer for this constructive suggestion. We fully agree that both cell-type specificity and functional validation are essential for optogenetic experiments. Accordingly, we performed two additional validation experiments, and all corresponding results have been included in **Figure EV1**.

1) we performed immunofluorescence colocalization analyses between the fluorescent reporters of the optogenetic constructs (ChR2-EYFP and NpHR-EYFP) and a glutamatergic neuronal marker. These results confirmed that both optogenetic tools were selectively expressed in ACC glutamatergic neurons, demonstrating the specificity of our viral targeting strategy.
2) we performed ex vivo whole-cell patch-clamp recordings to evaluate the functional efficacy of optogenetic activation. Light stimulation reliably evoked inward photocurrents and action potential firing in ChR2-expressing neurons, confirming the effectiveness of optogenetic activation.

These additional validation experiments further strengthen the specificity and reliability of the optogenetic manipulations used throughout this study.

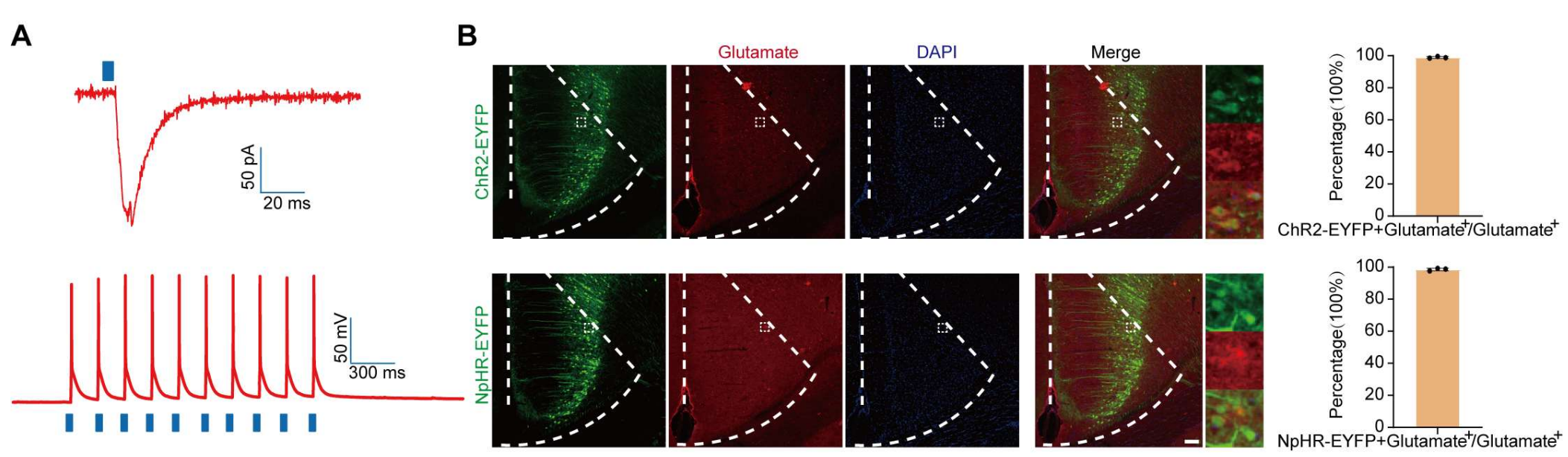

6. The study confirms that AK7 upregulation in ACC glutamatergic neurons modulates neuronal calcium activity and pain-related behaviors, but the direct molecular mechanism by which AK7 regulates neuronal activity remains largely unclear. As an adenylate kinase involved in nucleotide metabolism, whether AK7 upregulation alters the levels of ATP/ADP/AMP in the ACC region

needs to be experimentally verified. Furthermore, the authors should further investigate whether AK7 affects glutamatergic synaptic transmission or ion channel function by regulating cellular energy metabolism, and supplement relevant in vitro and in vivo experimental evidence to elucidate the specific molecular cascade of AK7 in pain modulation.

Response: We sincerely thank the reviewer for this insightful comment. We agree that elucidating the molecular mechanism by which AK7 regulates neuronal activity is essential for strengthening the mechanistic significance of this study. In response to this suggestion, we performed a series of additional experiments to evaluate key indicators of cellular energy metabolism in the ACC, and these new data have been incorporated into the revised manuscript as **Figures 5O-Q**.

Specifically, we measured the ATP/ADP ratio, adenosine levels, and the expression of phosphorylated AMPK (p-AMPK), a widely recognized indicator of cellular energy stress. Our results showed that CFA-induced chronic inflammatory pain was associated with a reduced ATP/ADP ratio, elevated adenosine levels, and increased p-AMPK expression in the ACC. Importantly, these metabolic alterations were significantly ameliorated following AK7 knockdown. Together, these findings suggest that AK7 may regulate neuronal function, at least in part, through disruption of nucleotide homeostasis and cellular energy metabolism, accompanied by activation of the AMPK-associated energy stress pathway. Although the precise downstream molecular cascade linking metabolic alterations to neuronal excitability remains to be fully elucidated, the newly added data substantially strengthen the mechanistic framework of the present study. In addition, we have expanded the Discussion section (Lines 732–745, highlighted in red) to discuss this potential metabolic regulatory mechanism and its limitations.

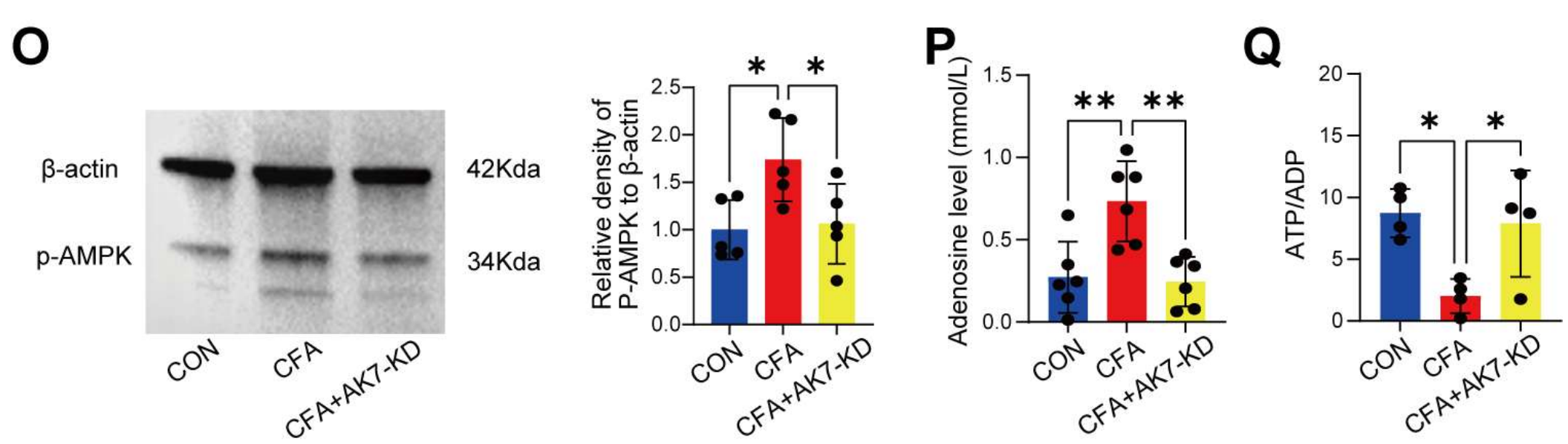

1st Revision - Editorial Decision 4th Aug 2026

Dear Dr. Li

Thank you for the submission of your revised manuscript. We have now received the enclosed reports from the referees and I am happy to say that all support its publication now. Referee 2 has 2 more suggestions that I would like you to address before we can proceed with the official acceptance of your manuscript. Please let me know if you cannot address these last 2 points so that we can discuss this further.

A few editorial requests will also need to be addressed:

- Please correct the conflict of interest subheading to "Disclosure and Competing Interests Statement"

- Please correct the author name discrepancy: Ru-Yu Yan in the ms vs. Ru-Yu Ran in our online submission system

- The author credits need to be removed from the ms file. All credits need to be entered during online ms submission.

- The REFERENCE format is not correct: It needs to be alphabetical, not numerical; et al needs to be used after 10 author names. Please use the EMBO reports reference format.

- The author checklist has not been completed, the entire column with pull down menus is missing entries/choices. Please send us a complete checklist with your final ms.

- The FUNDING INFO in our online system and in the ms file needs to be congruent. Currently, the following is missing in the system: Zhangjiagang Technology Project for Youth (ZJGQNKJ202424); the system has only one separate entry, while the rest of the funding is provided in the Comments box and is missing in the Acknowledgments - it should be removed from the box and added separately in the system and should be acknowledged in the ms: Suzhou Municipal Health Commission General Project (MSXM2024045), Zhangjiagang Science and Technology Plan Project (ZKYL2430) and Postdoctoral Fellowship Program of CPSF under Grant Number GZC20231890.

- The Materials and Methods section should include a separate Reagents and Tools Table file (listing key reagents, experimental models, software and relevant equipment and including their sources and relevant identifiers) and a Methods and Protocols section in which methods should be described using a step-by-step protocol format with bullet points. More information and a downloadable Table template is available in our guide to authors online: https://link.springer.com/journal/44319/submission-guidelines

- Please add the specific URL for your source data to the DAS (Data Availability Section)

- Materials and Methods should be just Methods.

- The manuscript sections should be in the following order: Title page - Abstract
- Introduction - Results - Discussion - Methods - Data Availability - Acknowledgments - Disclosure Statement & Competing Interests - References - Figure Legends - (Main Tables with legends if applicable) - Expanded View Figure Legends.

- The Ethics section should be in the Methods.

- Our routine image analysis detected a possible reuse between Figure 5W and Figure 6N. Can you please explain/clarify what happened?

* Figure Legends - Comments *

- 1. Please note that the panels for figure 7; is missing, although legends are provided in the manuscript. This needs to be rectified.
- Please note that the exact p values are not provided in the legends of figures 1C,E,F,G,H,I,K,M,N,P,Q; 2E,F,I,J,K,L; 3D,I,J,L,M,O,P; 4F,G,H; 5B,C,G,I,K,L,N,O,P,Q,S,T,U,X; 6B,F,G,I,J,L,P,Q,S,T; EV-3C,D,E,F; EV-4B,C. Please provide exact values as reasonable.
- Please indicate the statistical test used for data analysis in the legends of figures 4B,D,E.
- Please note that information related to n is missing in the legends of figures 4B; EV-1B; EV-2.
- Please note that the error bars are not defined in the legends of figures 1C,E,F,G,H,I,K,M,N,P,Q; 2A,E,F; 3B,D,E,I,J,L,M,O,P; 4F,G,H,I,J,K,L; 5B,C,E,F,G,I,K,L,N,O,P,Q,S,T,U,X; 6B,F,G,L,P,Q; EV-1B; EV-2; EV-4B,C.

I would like to suggest a few minor changes to the abstract that needs to be written in present tense. Please let me know if you

agree with this:

Chronic inflammation pain is a complex pathological condition involving multiple mechanisms in both the peripheral and central nervous systems. Growing evidence indicates that an imbalance in central nervous system function plays a crucial role in the progression of chronic pain, yet the underlying neural circuits and molecular targets remain largely unclear. Here we identify the anterior cingulate cortex (ACC) as a key mediator in chronic inflammation pain. Noxious stimulation increased c-Fos expression and calcium activity in both the ACC and the ventral tegmental area (VTA). Upregulation of adenosine kinase 7 (AK7) in ACC glutamatergic neurons emerges as an important factor in pain pathogenesis: interfering with AK7 expression reduces neuronal activity in the ACC and VTA and alleviates pain-related behaviors, while overexpressing AK7 enhances neuronal activity in these regions and exacerbates pain. Further experiments demonstrate that modulating the activity of VTA glutamatergic neurons can correspondingly reverse the effects of AK7 manipulation on pain phenotypes. In summary, our findings demonstrate that upregulation of AK7 in ACC promotes chronic pain through the ACC-VTA neural circuit.

EMBO press papers are accompanied online by A) a short (1-2 sentences) summary of the findings and their significance, B) 2-3 bullet points highlighting key results and C) a synopsis image that is exactly 550 pixels wide and 300 pixels high. The synopsis image should provide a sketch of the major findings, like a graphical abstract. Please note that text needs to be readable at the final size. Please send us this information along with the final manuscript.

I look forward to seeing a final version of your manuscript as soon as possible.

Best regards,
Esther

Esther Schnapp, PhD
Senior Editor
EMBO reports

Referee #1:

The authors have carefully revised the manuscript in response to the comments and have adequately addressed the concerns raised during the initial review. The revised manuscript has been substantially improved in terms of clarity, presentation, and scientific interpretation. I have no further major concerns and recommend acceptance for publication in EMBO Reports.

Referee #2:

The authors have provided a thorough and thoughtful response to the reviewers' comments and have made substantial efforts to improve the manuscript by incorporating additional experiments, new analyses, and a more comprehensive discussion of the relevant literature. Overall, the study has been significantly strengthened. However, two minor issues remain that should be addressed to further enhance the manuscript.

1.The manuscript does not specify at which time point(s) after pain model induction the electrophysiological experiments were performed. Because ACC neuronal activity evolves dynamically during the progression of chronic pain, the exact experimental time points should be clearly stated. This information is essential for accurately interpreting the physiological findings.
2.The VTA is the principal source of dopaminergic neurons and plays a pivotal role in chronic pain modulation. The manuscript would benefit from a more direct assessment of dopaminergic signaling during AK7 manipulation in the ACC-VTA circuit. Specifically, it remains unclear whether the analgesic effect produced by manipulating AK7 expression in ACC glutamatergic neurons is associated with enhanced dopamine release in downstream targets. If technically feasible, direct measurements of dopamine release (e.g., fiber photometry using genetically encoded dopamine sensors, GRAB-DA/dLight, carbon fiber electrodes (CFEs), or microdialysis) would substantially strengthen the mechanistic conclusions.

Referee #3:

The revised manuscript has significantly improved and fully meets the standards for publication.

Referee #1:

The authors have carefully revised the manuscript in response to the comments and have adequately addressed the concerns raised during the initial review. The revised manuscript has been substantially improved in terms of clarity, presentation, and scientific interpretation. I have no further major concerns and recommend acceptance for publication in EMBO Reports.

Response: Thank you very much for the positive comments and constructive suggestions.

Referee #2:

The authors have provided a thorough and thoughtful response to the reviewers' comments and have made substantial efforts to improve the manuscript by incorporating additional experiments, new analyses, and a more comprehensive discussion of the relevant literature. Overall, the study has been significantly strengthened. However, two minor issues remain that should be addressed to further enhance the manuscript.

1.The manuscript does not specify at which time point(s) after pain model induction the electrophysiological experiments were performed. Because ACC neuronal activity evolves dynamically during the progression of chronic pain, the exact experimental time points should be clearly stated. This information is essential for accurately interpreting the physiological findings.

Response: Thank you for the comment. In the study, electrophysiological recordings were performed on the third day following CFA injection. We have now clarified this time point in the Methods section of the revised manuscript (Lines 682–685, highlighted in red).

2.The VTA is the principal source of dopaminergic neurons and plays a pivotal role in chronic pain modulation. The manuscript would benefit from a more direct assessment of dopaminergic signaling during AK7 manipulation in the ACC-VTA circuit. Specifically, it remains unclear whether the analgesic effect produced by manipulating AK7 expression in ACC glutamatergic neurons is associated with enhanced dopamine release in downstream targets. If technically feasible, direct measurements of dopamine release (e.g., fiber photometry using genetically encoded dopamine sensors, GRAB-DA/dLight, carbon fiber electrodes (CFEs), or microdialysis) would substantially strengthen the mechanistic conclusions.

Response: We fully agree with the reviewer that clarifying whether the

analgesic effect induced by knockdown of AK7 expression is related to VTA dopaminergic neurons would significantly strengthen the mechanistic insights of our study. Previous studies have provided strong evidence that the ACC^{Glu}–VTA^{GABA}–VTA^{DA} circuit mediates pain (Nat Neurosci., 2024, PMID: 38172439), suggesting that VTA dopaminergic neurons may play a similar role in our model. Moreover, our staining data further indicate that over 10% of neurons in the VTA that receive ACC projections are dopaminergic, which also offers some support for the potential involvement of VTA dopaminergic neurons in pain modulation. Taken together, we speculate that the analgesic effect resulting from manipulation of AK7 in the ACC is mediated, at least in part, through VTA dopaminergic neurons. We plan to further investigate this possibility in future studies using approaches such as dopamine sensors and microdialysis. The above description has been incorporated into the Discussion section of the revised manuscript (Lines 447–480, highlighted in red).

Referee #3:

The revised manuscript has significantly improved and fully meets the standards for publication.

Response: We are very grateful for the reviewer’s positive comments.

Dr. Yong-Chang Li
Soochow University
iangsu Key Laboratory of Neuropsychiatric Diseases and Institute of Neuroscience
China

Dear Dr. Li,

Since my colleague Esther Schnapp is currently traveling, I have taken over the handling of your manuscript. Former referee #2 was asked to assess your point-by-point response and now supports publication. Thank you also for taking care of the remaining editorial points. I am thus very pleased to accept your manuscript for publication in the next available issue of EMBO reports. Thank you for your contribution to our journal.

Your manuscript will be processed for publication by EMBO Press. It will be copy edited and you will receive page proofs prior to publication. Please note that you will be contacted by Springer Nature Author Services to complete licensing and payment information.

You may qualify for financial assistance for your publication charges - either via a Springer Nature fully open access agreement or an EMBO initiative. Check your eligibility: https://link.springer.com/journal/44319/how-to-publish-with-us

Should you be planning a Press Release on your article, please notify the production team via this form in order to coordinate publication and release dates: https://forms.cloud.microsoft/e/eH4A8JVP6g

If you have any questions, please do not hesitate to contact the Editorial Office. Thank you for your contribution to EMBO Reports.

Yours sincerely,

Martina Rembold, PhD
Senior Editor
EMBO reports

>>> Please note that it is EMBO Reports policy for the transcript of the editorial process (containing referee reports and your response letter) to be published as an online supplement to each paper. If you do NOT want this, you will need to inform the Editorial Office via email immediately. More information is available here: https://link.springer.com/partners/embo-press/editorial-policies#Peer%20review

EMBO Press Author Checklist

Corresponding Author Name: Ying Cao, Xiao-Dong Xu, Yong-Chang Li	
Journal Submitted to: EMBO Reports	
Manuscript Number: EMBOR-2026-63646V1	

USEFUL LINKS FOR COMPLETING THIS FORM
The EMBO Journal - Author Guidelines
EMBO Reports - Author Guidelines
Molecular Systems Biology - Author Guidelines
EMBO Molecular Medicine - Author Guidelines

Reporting Checklist for Life Science Articles (updated January
This checklist is adapted from Materials Design Analysis Reporting (MDAR) Checklist for Authors. MDAR establishes a minimum set of requirements in transparent reporting in the life sciences (see Statement of Task: 10.31222/osf.io/9sm4x). Please follow the journal's guidelines in preparing your
Please note that a copy of this checklist will be published alongside your article.

Abridged guidelines for figures
1. Data
The data shown in figures should satisfy the following conditions:

- the data were obtained and processed according to the field's best practice and are presented to reflect the results of the experiments in an accurate and unbiased manner.
- ideally, figure panels should include only measurements that are directly comparable to each other and obtained with the same assay.
- plots include clearly labeled error bars for independent experiments and sample sizes. Unless justified, error bars should not be shown for technical
- if n<5, the individual data points from each experiment should be plotted. Any statistical test employed should be justified.
- Source Data should be included to report the data underlying figures according to the guidelines set out in the authorship guidelines on Data

2. Captions
Each figure caption should contain the following information, for each panel where they are relevant:

- a specification of the experimental system investigated (eg cell line, species name).
- the assay(s) and method(s) used to carry out the reported observations and measurements.
- an explicit mention of the biological and chemical entity(ies) that are being measured.
- an explicit mention of the biological and chemical entity(ies) that are altered/varied/perturbed in a controlled manner.
- the exact sample size (n) for each experimental group/condition, given as a number, not a range;
- a description of the sample collection allowing the reader to understand whether the samples represent technical or biological replicates (including how many animals, litters, cultures, etc.).
- a statement of how many times the experiment shown was independently replicated in the laboratory.
- definitions of statistical methods and measures:
  - common tests, such as t-test (please specify whether paired vs. unpaired), simple χ^2 tests, Wilcoxon and Mann-Whitney tests, can be unambiguously identified by name only, but more complex techniques should be described in the methods section;
  - are tests one-sided or two-sided?
  - are there adjustments for multiple comparisons?
  - exact statistical test results, e.g., P values = x but not P values < x;
  - definition of 'center values' as median or average;
  - definition of error bars as s.d. or s.e.m.

Please complete ALL of the questions below.
Select "Not Applicable" only when the requested information is not relevant for your study.

Materials

Newly Created Materials	Information included in the manuscript?	In which section is the information available? (Reagents and Tools Table, Materials and Methods, Figures, Data Availability Section)
New materials and reagents need to be available; do any restrictions apply?	Not Applicable	

Antibodies	Information included in the manuscript?	In which section is the information available? (Reagents and Tools Table, Materials and Methods, Figures, Data Availability Section)
For **antibodies** provide the following information:<br>- Commercial antibodies: RRID (if possible) or supplier name, catalogue number and or/clone number<br>- Non-commercial: RRID or citation	Yes	Materials and Methords Section

DNA and RNA sequences	Information included in the manuscript?	In which section is the information available? (Reagents and Tools Table, Materials and Methods, Figures, Data Availability Section)
**Short novel DNA or RNA including primers, probes**: provide the sequences.	Yes	Materials and Methords Section

Cell materials	Information included in the manuscript?	In which section is the information available? (Reagents and Tools Table, Materials and Methods, Figures, Data Availability Section)
**Cell lines:** Provide species information, strain. Provide accession number in repository **OR** supplier name, catalog number, clone number, and/**OR** RRID.	Not Applicable	
**Primary cultures:** Provide species, strain, sex of origin, genetic modification status.	Not Applicable	
Report if the cell lines were recently **authenticated** (e.g., by STR profiling) and tested for mycoplasma contamination.	Not Applicable	

Experimental animals	Information included in the manuscript?	In which section is the information available? (Reagents and Tools Table, Materials and Methods, Figures, Data Availability Section)
**Laboratory animals or Model organisms:** Provide species, strain, sex, age, genetic modification status. Provide accession number in repository **OR** supplier name, catalog number, clone number, **OR** RRID.	Yes	Materials and Methords Section
**Animal observed in or captured from the field:** Provide species, sex, and age where possible.	Yes	Materials and Methords Section
Please detail **housing and husbandry conditions**.	Yes	Materials and Methords Section

Plants and microbes	Information included in the manuscript?	In which section is the information available? (Reagents and Tools Table, Materials and Methods, Figures, Data Availability Section)
**Plants:** provide species and strain, ecotype and cultivar where relevant, unique accession number if available, and source (including location for collected wild specimens).	Not Applicable	
**Microbes:** provide species and strain, unique accession number if available, and source.	Not Applicable	

Human research participants	Information included in the manuscript?	In which section is the information available? (Reagents and Tools Table, Materials and Methods, Figures, Data Availability Section)
If collected and within the bounds of privacy constraints report on age, sex and gender or ethnicity for all study participants.	Not Applicable	

Core facilities	Information included in the manuscript?	In which section is the information available? (Reagents and Tools Table, Materials and Methods, Figures, Data Availability Section)
If your work benefited from core facilities, was their service mentioned in the acknowledgments section?	Not Applicable	

Design

Study protocol	Information included in the manuscript?	In which section is the information available? (Reagents and Tools Table, Materials and Methods, Figures, Data Availability Section)
If study protocol has been **pre-registered, provide DOI in the manuscript**. For clinical trials, provide the trial registration number **OR** cite DOI.	Not Applicable	
Report the **clinical trial registration number** (at ClinicalTrials.gov or equivalent), where applicable.	Not Applicable	

Laboratory protocol	Information included in the manuscript?	In which section is the information available? (Reagents and Tools Table, Materials and Methods, Figures, Data Availability Section)
Provide DOI OR other citation details if **external detailed step-by-step protocols** are available.	Not Applicable	Materials and Methords Section

Experimental study design and statistics	Information included in the manuscript?	In which section is the information available? (Reagents and Tools Table, Materials and Methods, Figures, Data Availability Section)
Include a statement about **sample size** estimate even if no statistical methods were used.	Not Applicable	
Were any steps taken to minimize the effects of subjective bias when allocating animals/samples to treatment (e.g. **randomization procedure**)? If yes, have they been described?	Not Applicable	
Include a statement about **blinding** even if no blinding was done.	Not Applicable	
Describe **inclusion/exclusion criteria** if samples or animals were excluded from the analysis. Were the criteria pre-established?<br><br>If sample or data points were omitted from analysis, report if this was due to attrition or intentional exclusion and provide justification.	Not Applicable	
For every figure, are **statistical tests** justified as appropriate? Do the data meet the assumptions of the tests (e.g., normal distribution)? Describe any methods used to assess it. Is there an estimate of variation within each group of data? Is the variance similar between the groups that are being statistically compared?	Not Applicable	Materials and Methords Section

Sample definition and in-laboratory replication	Information included in the manuscript?	In which section is the information available? (Reagents and Tools Table, Materials and Methods, Figures, Data Availability Section)
In the figure legends: state number of times the experiment was **replicated** in laboratory.	Yes	Figure legends
In the figure legends: define whether data describe **technical or biological replicates**.	Not Applicable	

Ethics

Ethics	Information included in the manuscript?	In which section is the information available? (Reagents and Tools Table, Materials and Methods, Figures, Data Availability Section)
Studies involving **human participants**: State details of **authority granting ethics approval** (IRB or equivalent committee(s), provide reference number for approval.	Not Applicable	
Studies involving **human participants**: Include a statement confirming that **informed consent** was obtained from all subjects and that the experiments conformed to the principles set out in the WMA Declaration of Helsinki and the Department of Health and Human Services Belmont Report.	Not Applicable	
Studies involving **human participants:** For publication of **patient photos**, include a statement confirming that consent to publish was obtained.	Not Applicable	
Studies involving experimental **animals**: State details of **authority granting ethics approval** (IRB or equivalent committee(s), provide reference number for approval. Include a statement of compliance with ethical regulations.	Yes	Materials and Methods Section
Studies involving **specimen and field samples:** State if relevant **permits** obtained, provide details of authority approving study; if none were required, explain why.	Not Applicable	

Dual Use Research of Concern (DURC)	Information included in the manuscript?	In which section is the information available? (Reagents and Tools Table, Materials and Methods, Figures, Data Availability Section)
Could your study fall under dual use research restrictions? Please check biosecurity documents and list of **select agents and toxins** (CDC): https://www.selectagents.gov/sat/list.htm	Not Applicable	
If you used a select agent, is the security level of the lab appropriate and reported in the manuscript?	Not Applicable	
If a study is subject to dual use research of concern regulations, is the name of the **authority granting approval and reference number** for the regulatory approval provided in the manuscript?	Not Applicable	

Reporting

The MDAR framework recommends adoption of discipline-specific guidelines, established and endorsed through community initiatives. Journals have their own policy about requiring specific guidelines and recommendations to complement MDAR.

Adherence to community standards	Information included in the manuscript?	In which section is the information available? (Reagents and Tools Table, Materials and Methods, Figures, Data Availability Section)
State if relevant guidelines or checklists (e.g., **ICMJE, MIBBI, ARRIVE, PRISMA**) have been followed or provided.	Not Applicable	
For **tumor marker prognostic studies,** we recommend that you follow the **REMARK** reporting guidelines (see link list at top right). See author guidelines, under 'Reporting Guidelines'. Please confirm you have followed these guidelines.	Not Applicable	
For **phase II and III randomized controlled trials**, please refer to the **CONSORT** flow diagram (see link list at top right) and submit the CONSORT checklist (see link list at top right) with your submission. See author guidelines, under 'Reporting Guidelines'. Please confirm you have submitted this list.	Not Applicable	

Data Availability

Data availability	Information included in the manuscript?	In which section is the information available? (Reagents and Tools Table, Materials and Methods, Figures, Data Availability Section)
Have **primary datasets** been deposited according to the journal's guidelines (see 'Data Deposition' section) and the respective accession numbers provided in the Data Availability Section?	Yes	Data Availability Section
Were **human clinical and genomic datasets** deposited in a public access-controlled repository in accordance to ethical obligations to the patients and to the applicable consent agreement?	Not Applicable	
Are **computational models** that are central and integral to a study available without restrictions in a machine-readable form? Were the relevant accession numbers or links provided?	Not Applicable	
If publicly available data were reused, provide the respective **data citations in the reference list**.	Not Applicable	
