# Supplementary material for: Upregulation of AK7 in ACC-VTA glutamatergic projections contributes to chronic inflammation pain in male mice
Source: EMBO Rep. 2026 Sep 21;27(20):5860–80. doi: 10.1038/s44319-026-00940-z (PMC13694706; doi:10.1038/s44319-026-00940-z)
Supplement: Supplementary file 2 [file 44319_2026_940_MOESM2_ESM.pdf]

Expanded View Figures

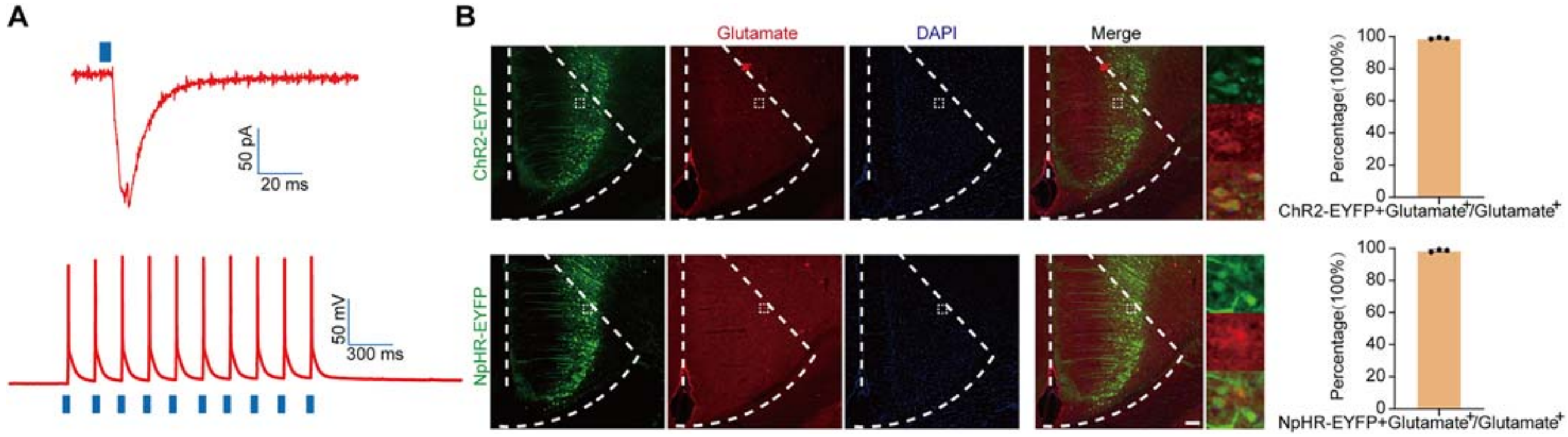

Figure EV1. Functional validation and cell-type specificity of optogenetic viral expression in ACC glutamatergic neurons.

(**A**) Ex vivo whole-cell patch-clamp recordings demonstrating the functional efficacy of ChR2 expressed in ACC glutamatergic neurons. Upper panel: Representative light-evoked inward photocurrent recorded from a ChR2-expressing neuron following brief blue light stimulation. Lower panel: Representative action potential firing induced by repetitive blue light pulses in a ChR2-expressing neuron. Scale bars: 50 pA, 20 ms (upper); 50 mV, 300 ms (lower). (**B**) Representative immunofluorescence images showing colocalization of ChR2-EYFP (upper panels) or NpHR-EYFP (lower panels) with the glutamatergic neuronal marker Glutamate in the ACC ($n = 3$ mice per group). Scale bar = 100 µm. The data are represented as mean ± SEM.

© The Author(s)

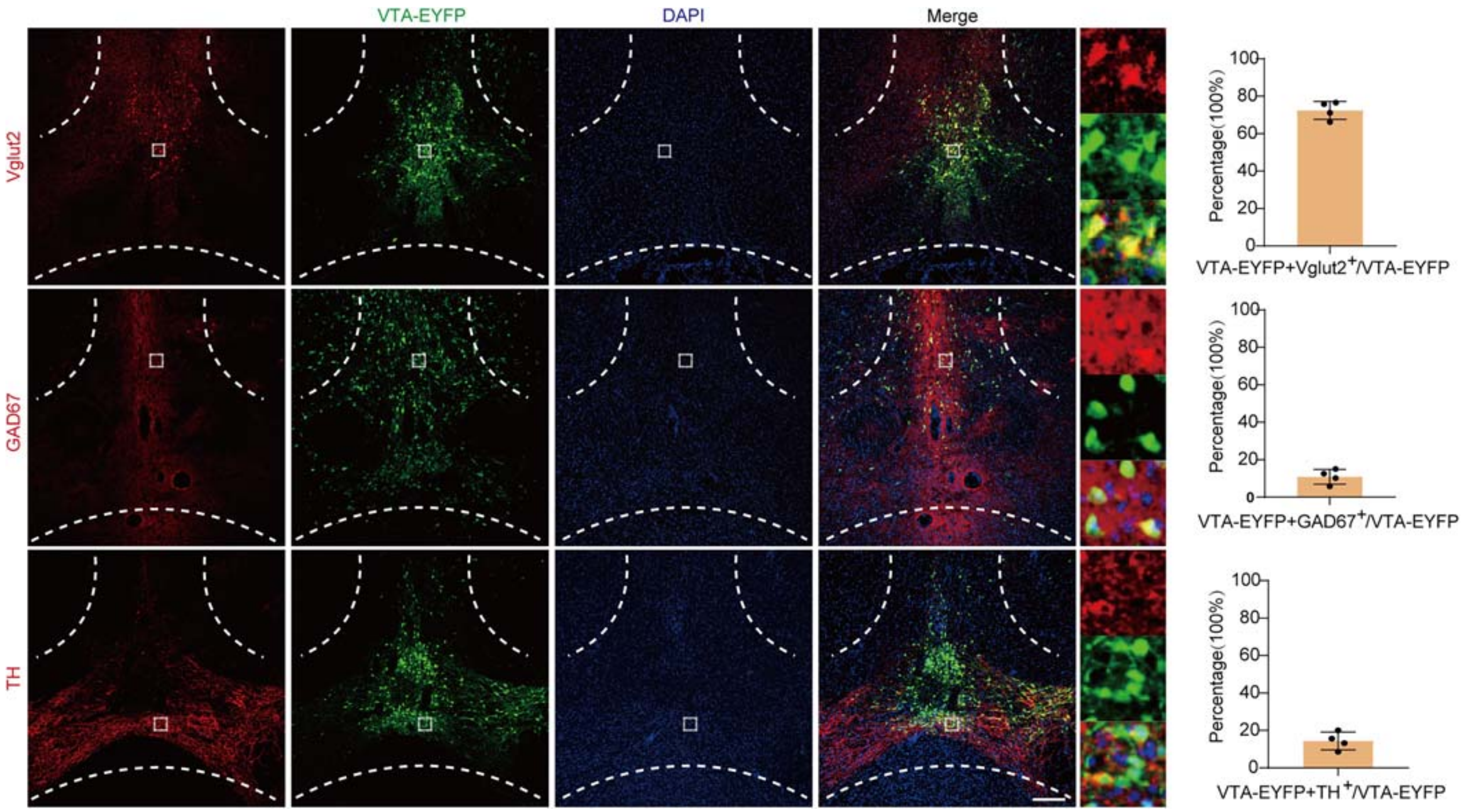

Figure EV2. Identification of the major postsynaptic neuronal population receiving ACC glutamatergic projections in the VTA.

A dual-virus anterograde tracing strategy was used to label ACC-derived EYFP-positive projections in the VTA, followed by immunofluorescence colocalization analysis with markers for the three major neuronal populations in the VTA: VGLUT2 (glutamatergic neurons, upper panels), GAD67 (GABAergic neurons, middle panels), and TH (dopaminergic neurons, lower panels) ($n = 4$ mice per group). Scale bar = 100 µm. The data are represented as mean ± SEM.

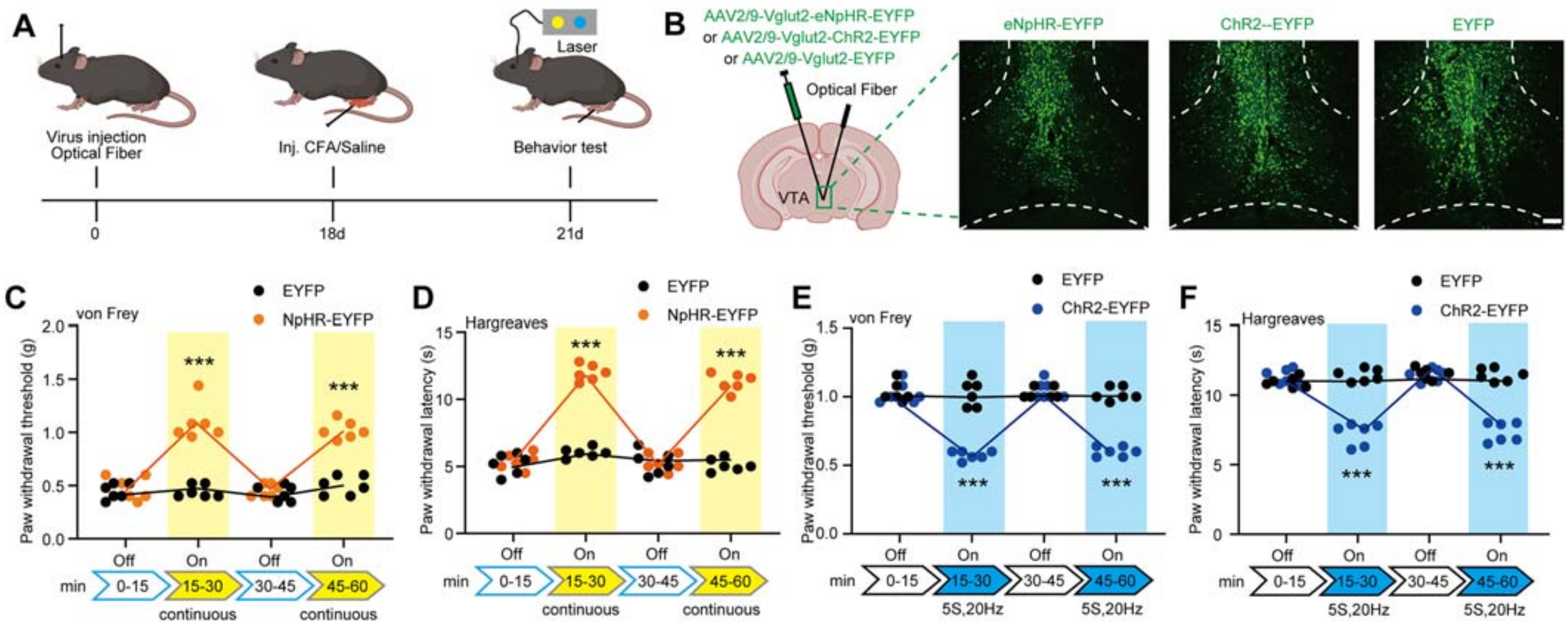

Figure EV3. Optogenetic manipulation of VTA glutamatergic neurons alters pain behavior.

(**A**) Experimental design for optogenetic manipulation of VTA glutamatergic neurons. (**B**) Representative images showing expression of eNpHR or ChR2 in VTA glutamatergic neurons. (**C**, **D**) Optogenetic inhibition of VTA glutamatergic neurons significantly increased PWT and PWL in CFA mice (***$P < 0.0001$, two-way ANOVA followed by Sidak's multiple-comparisons test, $n = 6$ mice per group). (**E**, **F**) Optogenetic activation of VTA glutamatergic neurons decreased PWT and PWL in CON mice (***$P < 0.0001$, two-way ANOVA followed by Sidak's multiple-comparisons test, $n = 6$ mice per group). Scale bar = 100 µm. The data are represented as mean ± SEM.

 © The Author(s)

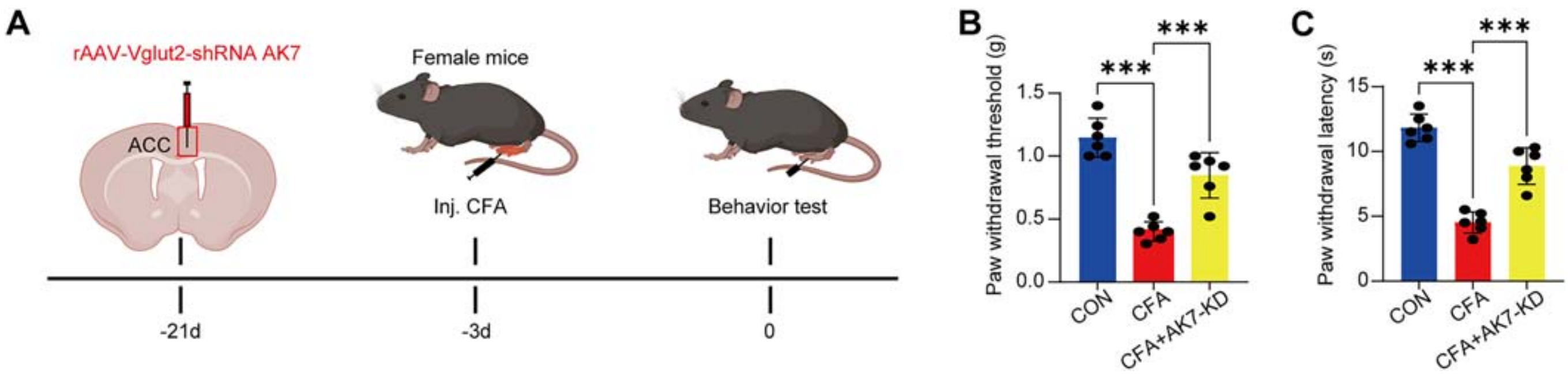

Figure EV4. AK7 knockdown in ACC glutamatergic neurons attenuates CFA-induced mechanical and thermal hypersensitivity in female mice.

(**A**) Schematic illustration of stereotaxic viral delivery for selective knockdown of AK7 in ACC glutamatergic neurons of female mice. (**B**, **C**) Knockdown of AK7 significantly increased PWT and PWL in CFA mice (***$P < 0.0001$, one-way ANOVA with Tukey's multiple-comparisons test, $n = 6$ mice per group). The data are represented as mean ± SEM.
